# USP7 depletion potentiates HIF2α degradation and inhibits clear cell renal cell carcinoma progression

**DOI:** 10.1038/s41419-024-07136-0

**Published:** 2024-10-15

**Authors:** Rongfu Tu, Junpeng Ma, Yule Chen, Ye Kang, Doudou Ren, Zeqiong Cai, Ru Zhang, Yiwen Pan, Yijia Liu, Yanyan Da, Yao Xu, Yahuan Yu, Donghai Wang, Jingchao Wang, Yang Dong, Xinlan Lu, Chengsheng Zhang

**Affiliations:** 1 The First Affiliated Hospital of Xi’an Jiaotong University, Center for Precision Cancer Medicine, MED-X Institute, 710000 Xi’an, China; 2 The First Affiliated Hospital, Jiangxi Medical College, Nanchang University, Center for Molecular Diagnosis and Precision Medicine, 1519 Dongyue Dadao, 330209 Nanchang, China; 3 The First Affiliated Hospital, Jiangxi Medical College, Nanchang University, Department of Clinical Laboratory, 1519 Dongyue Dadao, 330209 Nanchang, China; 4 The First Affiliated Hospital, Jiangxi Medical College, Nanchang University, Jiangxi Provincial Center for Advanced Diagnostic Technology and Precision Medicine, 1519 DongYue Dadao, 330209 Nanchang, China; 5 Department of Urology, The First Affiliated Hospital of Xi’an Jiaotong University, 710000 Xi’an, China; 6 The First Affiliated Hospital of Xi’an Jiaotong University, Precision Medicine Center, 710000 Xi’an, China; 7 Department of Nephrology, The Affiliated Hospital of Qingdao University, 266100 Qingdao, China; 8 Frontier Science Center for Immunology and Metabolism, Medical Research Institute, Wuhan University, 430071 Wuhan, China; 9 Guangdong Key Laboratory of Genome Instability and Human Disease Prevention, Department of Biochemistry and Molecular Biology, Shenzhen University School of Medicine, 518055 Shenzhen, China; 10 Department of Pathology, Zhongnan Hospital of Wuhan University, 430071 Wuhan, China; 11 Department of Gastroenterology, The First Affiliated Hospital of Xi’an Jiaotong University, 710000 Xi’an, China; 12 Department of Medical Genetics, The First Affiliated Hospital, Jiangxi Medical College, Nanchang University, 1519 DongYue Dadao, 330209 Nanchang, China

**Keywords:** Renal cell carcinoma, Oncogenesis

## Abstract

Clear cell renal cell carcinoma (ccRCC) is characterized by *Von Hippel Lindau* (*VHL*) gene loss of function mutation, which leads to the accumulation of hypoxia-inducible factor 2α (HIF2α). HIF2α has been well-established as one of the major oncogenic drivers of ccRCC, however, its therapeutic targeting remains a challenge. Through an analysis of proteomic data from ccRCCs and adjacent non-tumor tissues, we herein revealed that Ubiquitin-Specific Peptidase 7 (USP7) was upregulated in tumor tissues, and its depletion by inhibitors or shRNAs caused significant suppression of tumor progression in vitro and in vivo. Mechanistically, USP7 expression is activated by the transcription factors FUBP1 and FUBP3, and it promotes tumor progression mainly by deubiquitinating and stabilizing HIF2α. Moreover, the combination of USP7 inhibitors and afatinib (an ERBB family inhibitor) coordinately induce cell death and tumor suppression. In mechanism, afatinib indirectly inhibits USP7 transcription and accelerates the degradation of HIF2α protein, and the combination of them caused a more profound suppression of HIF2α abundance. These findings reveal a FUBPs-USP7-HIF2α regulatory axis that underlies the progression of ccRCC and provides a rationale for therapeutic targeting of oncogenic HIF2α via combinational treatment of USP7 inhibitor and afatinib.

## Introduction

Renal cell carcinoma (RCC) is the most common and lethal tumor of the urological system, which affects over 400,000 individuals worldwide each year [1]. ~75% of RCC patients are diagnosed as ccRCC [2, 3]. Although the early stage of ccRCC can be successfully treated with surgical strategies, up to a third of patients will develop metastatic ccRCC, which is almost uniformly lethal [3]. Nearly all ccRCC tumors contain inactivating mutations of the gene *VHL*, which encodes the pVHL protein, a part of an E3 ubiquitin ligase that has a fundamental role in oxygen sensing by targeting the α-subunit of hypoxia-inducible factor (HIF) for degradation under normoxic conditions [4, 5]. *VHL* mutation leads to the accumulation of HIF1α and/or HIF2α even in normoxia [6], however, it has been well-established that HIF2α, but not HIF1α, is the critical oncogenic substrate of VHL in ccRCC [7–9].

HIF2α is deeply involved in angiogenesis and multiple other processes, and targeting HIF2α has been considered a promising therapeutic strategy for ccRCC [10, 11]. A unique 290-Å cavity in the PAS-B domain of HIF2α was identified, which paved the way for the identification of small molecule inhibitors that allosterically disrupt its heterodimerization with HIF1β [12–14]. However, these inhibitors were designed to inhibit HIF2α transcriptional activity and could only inhibit the ccRCC progression in certain preclinical models [15, 16], and the only approved such drug Belzutifan (PT2977) with only an overall response rate of 49% (95% CI: 36–62) in *VHL* mutant ccRCC [17]. Development of inhibitors directly targeting HIF2α stabilization has been a challenge for decades owing to the “undruggable” protein structure of transcription factors. Therefore, it remains critical to identify the key pathway regulating HIF2α protein stability, which may become a novel therapeutic target.

Deubiquitinase (DUB) has been reported to be deeply involved in human diseases, especially cancers, and targeting deubiquitinase provides emerging opportunities for cancer treatment [18]. Recently, USP13 was reported to promote ccRCC progression by deubiquitinating and stabilizing zinc fingers and homeoboxes 2 (ZHX2), which is another oncogenic substrate of pVHL [19, 20]. However, as for ccRCC, it remains less clear as to which deubiquitinase might represent a realistic therapeutic target. Deubiquitinase ubiquitin-specific protease 7 (USP7), also known as herpesvirus-associated ubiquitin-specific protease (HAUSP), has been regarded as an important regulator of tumorigenesis in several cancers [21], but its potential roles in ccRCC and the underlying mechanisms have long been mysterious.

Afatinib is an oral epidermal growth factor receptor tyrosine kinase inhibitor targeting the ERBB family (including EGFR, HER2, ERBB3, and ERBB4), and has been approved for the treatment of locally advanced or metastatic non-small-cell lung cancer (NSCLC) with an EGFR-sensitive mutation [22]. Due to the central role of the ERBB family in the tumorigenesis of solid tumors [23], afatinib is now in a phase II clinical trial (https://classic.clinicaltrials.gov/ct2/, NCT02465060) for the treatment of many solid tumors, including ccRCC. Two members of the ERBB family, EGFR and HER2, were reported to sustain HIF2α expression in breast cancer [24, 25], but the underlying mechanisms were not clear. In addition, the correlation between afatinib and oncogenic HIF2α is unknown.

We herein identified a sustained USP7 expression that, transcriptionally activated by FUBP1 and FUBP3, is essential for HIF2α stabilization and tumor progression in ccRCC. Moreover, we developed a mechanism-based therapeutic strategy for targeting oncogenic HIF2α via the combination of USP7 inhibitor and afatinib.

## Results

### USP7 is transcriptionally activated by FUBP1 and FUBP3 in ccRCCs

**Fig. 1:**
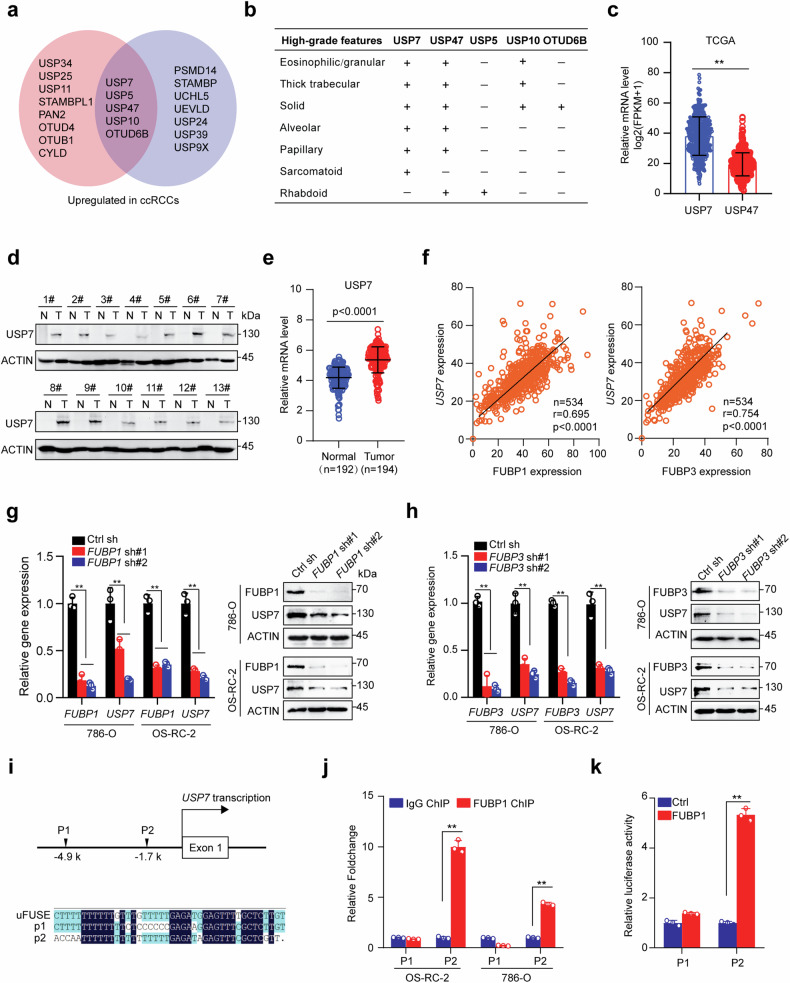
USP7 is transcriptionally activated by FUBP1 and FUBP3 in ccRCCs. **a** Venn diagrams showing up-regulated deubiquitinases in ccRCCs compared to adjacent normal tissues in two independent studies. The red diagram was identified in PMID: 35440542, and the blue one was identified in PMID: 36563681. **b** Comparison of five overlapped candidate protein levels in ccRCCs containing one of seven high-grade morphologic features and tumors without any of the aforementioned seven high-grade features, “+” represents the upregulation in certain subgroups, “−” represents no upregulation in certain subgroups. **c** The mRNA expression of USP7 and USP47 in 534 ccRCC samples was shown, and the data was obtained from the Cancer Genome Atlas Program (TCGA database, https://www.cancer.gov/). **d** USP7 protein expression in 13 ccRCCs and paired non-cancerous tissues were analyzed using Western blot. **e** Analysis of relative USP7 mRNA level in normal kidney (Normal) and ccRCC (Tumor) tissues using R2 genomics analysis and visualization platform (https://hgserver1.amc.nl/). **f** The mRNA correlation between FUBP1/FUBP3 and USP7 in 534 ccRCC samples was shown, the data was obtained from the Cancer Genome Atlas Program (TCGA database, https://www.cancer.gov/). **g** Cells were infected with control or FUBP1 shRNAs, FUBP1 and USP7 expression were analyzed using qRT-PCR and Western Blot. **h** Cells were infected with control or FUBP3 shRNAs, FUBP3 and USP7 expression were analyzed using qRT-PCR and Western Blot. **i** Schematic presentation of two potential FUBP1 binding sites proximal to the USP7 transcription initiation site, uFUSE: the binding sequence of FUBP1 within the USP29 promoter. **j** ChIP analysis of FUBP1 binding to the USP7 promoter in OS-RC-2 or 786-O cells. The binding signal was normalized to ACTIN and averages of fold enrichment between the FUBP1 antibodies and IgG are shown. **k** USP7 promoter sequence P1 (-5041bp - -4682bp) or P2 (-1860bp - -1501bp) was cloned into the pGL3-basic vector, Luciferase assays were carried out, and reporter activities are presented as average fold induction. The experiments were independently repeated three times with similar results, and the Graph shows mean ± SD from triplicates in one experiment (**g**, **h**, **j**, and **k**).

DUB has been regarded as a promising therapeutic target in cancers. In this study, we compared DUB family protein expression in ccRCC and the adjacent normal tissues using two published proteomics data [26, 27], and revealed that five DUBs (USP5, USP7, USP10, USP47, and OTUD6B) were commonly upregulated in ccRCCs (Fig. 1a). Seven high-grade morphologic features in ccRCCs were previously assessed and quantified as high-grade feature count (HGFC, range from 0-7) per tumor, and high HGFC (>3) was associated with a worse prognosis [27]. We compared the above five proteins’ expression in tumors with certain high-grade features and tumors without any high-grade features and found that USP7 and USP47 were upregulated in tumors with as many as six features (Fig. 1b), indicating that upregulation of USP7 and USP47 may become a biomarker for prognosis. We compared the expression levels of USP7 and USP47 in 534 ccRCC samples from the TCGA dataset and revealed that the abundance of USP7 (average log2(FPKM + 1) value 38.1) was much higher than that of USP47 (average log2(FPKM + 1) value 19.4) (Fig. 1c). Subsequently USP7 was chosen for the further study.

We first confirmed the upregulation of USP7 protein level in ccRCCs compared to the adjacent normal tissues (Fig. 1d). To decipher the molecular mechanisms underlying USP7 expression, we analyzed the mRNA expression of USP7 in ccRCCs and normal renal tissues. As expected, ccRCC tissues showed an upregulation of USP7 mRNA (Fig. 1e), arguing its transcriptional activation in tumors. We next performed in silico analysis of the correlation between USP7 and 795 transcription factors in 534 ccRCC samples, and the top 50 transcription factors positively correlated with USP7 were shown, including two members of the far upstream element binding protein (FUBP) family, FUBP1 and FUBP3, which were reported to have similar properties of DNA sequence binding and regulating gene expression (Fig. 1f, Supplementary Fig. S1a) [28]. While FUBP3 was not as overexpressed in tumors as FUBP1 (Supplementary Fig. S1b–d), both FUBP1 and FUBP3 depletion by shRNAs caused significant suppression of USP7 gene transcription (Fig. 1g and h). There are two putative FUBP1/FUBP3 binding elements within the USP7 promoter region (p1, -4.9 k; p2, -1.7 k) was found (Fig. 1i). Chromatin immunoprecipitation (ChIP) assay was performed in 786-O and OS-RC-2 cells using FUBP1 antibodies, which revealed a marked increase of FUBP1 recruitment in p2, but not in p1, in both cell lines (Fig. 1j). Luciferase reporter assays confirmed that FUBP1 significantly activated RE2 as compared with vector control or RE1 (Fig. 1k). Taken together, these findings suggest that FUBP1/FUBP3 may directly activate USP7 transcription in ccRCC cells.

### USP7 depletion suppresses *VHL* mutant ccRCC progression in vitro and in vivo

**Fig. 2:**
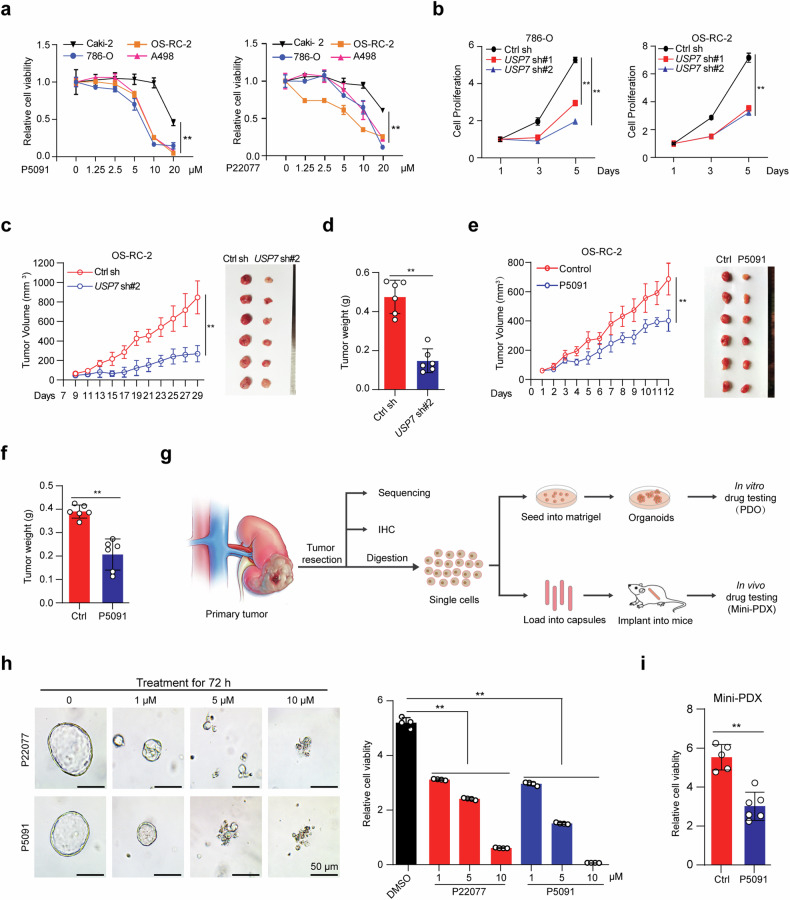
USP7 depletion suppresses *VHL* mutant ccRCC progression in vitro and in vivo. **a** ccRCC cells were seeded in 96-well plates, 12 h later, the cells were treated with the indicated concentration of P5091 or P22077 for 24 h, and cell viability was measured using CCK-8. **b** ccRCC cells with or without USP7 knockdown were seeded in 96-well plates, and cell proliferation was measured using CCK-8. **c** and **d** OS-RC-2 cells with or without USP7 knockdown were resuspended in 200 μL PBS containing 50% matrigel and subcutaneously injected into nude mice (*n* = 6 for each group), the tumor growth (**c**) and tumor weight (**d**) were analyzed. **e** and **f** OS-RC-2 xenograft tumor with or without treatment of P5091 (i.p. 25 mg/kg), tumor growth curve and tumor images (**e**), and tumor weight (**f**) from mice subjected to indicated treatments were shown (*n* = 6 for each group). **g** Diagram of the process of establishing *VHL* mutant ccRCC PDO and mini-PDX model from patient tumors for in vitro and in vivo drug treatment. **h** ccRCC PDOs were seeded in 96 well plates, 48 h later, PDOs were treated with the indicated concentration of P5091 or P22077 for 72 h, Representative PDO images were shown (left) and the cell viability was analyzed using CellTiter-Glo® Luminescent Cell Viability Assay (right). Scale bar, 50 μm. **i** Primary ccRCC cells were loaded into capsules and implanted into nude mice for constructing the mini-PDX model, mice were administered with saline or P5091(i.p. 25 mg/kg) for 7 days, relative tumor viability was detected using the CellTiter-Glo® Luminescent Cell Viability Assay. The experiments were independently repeated three times with similar results (**a**, **b**, and **h**). Data are shown in mean ± SD, **p < 0.01.

To explore the potential role of USP7 in ccRCC, we treated four ccRCC cell lines with specific USP7 inhibitors P5091 or P22077 and revealed that both inhibitors showed significant cytotoxicity. Moreover, *VHL* mutant ccRCC cell lines, including OS-RC-2, 786-O, and A498 seemed to be more sensitive to USP7 inhibition (Fig. 2a, Supplementary Fig. S2a–c). Considering the *VHL* gene is inactivated in more than 90% of ccRCCs [29], we then focused our study on *VHL* mutant ccRCC. Consistent with the results from inhibitors, the knockdown of USP7 by specific shRNAs also markedly suppressed the proliferation of 786-O and OS-RC-2 cells (Fig. 2b, Supplementary Fig. S2d). To evaluate whether USP7 is essential for tumor growth in vivo, we established the human renal cancer xenograft by subcutaneously injecting OS-RC-2 cells with or without *USP7* knockdown into nude mice. Consistent with the in vitro findings, USP7 knockdown suppressed the tumor growth in the mice (Fig. 2c, d). IHC analysis of Ki-67 and cleaved Caspase-3 demonstrated strong inhibition of cell proliferation and massive intratumoral cell death in the USP7-depleted cohort (Supplementary Fig. S2e). Treatment with USP7 inhibitor P5091 in the xenograft model showed similar tumor growth inhibition but had minimal effects on the mice’s body weight (Fig. 2e, f, Supplementary Fig. S2f, g).

Patient-derived organoid (PDO) and mini-patient-derived xenograft (Mini-PDX) models have emerged as robust and reliable tumor models for rapidly testing drug efficacy [30, 31]. We collected one human ccRCC sample, confirmed the *VHL* gene deficiency and HIF2α activation by genomic sequencing (data not shown) and IHC (Supplementary Fig. S2h), respectively, and then established the *VHL* mutant ccRCC PDO and Mini-PDX platforms (Fig. 2g). Single drug treatment with either P22077 or P5091 significantly inhibited the ccRCC PDO growth in a dose-depended manner (Fig. 2h), and P5091 treatment similarly suppressed the primary tumor cells growth in the Mini-PDX model (Fig. 2i). These findings showed that depletion of USP7 in ccRCC restrained tumor progression in vivo and in vitro, suggesting that USP7 may become a potential therapeutic target for ccRCC treatment.

### Knockdown of USP7 impairs the HIF2α transcriptional program

**Fig. 3:**
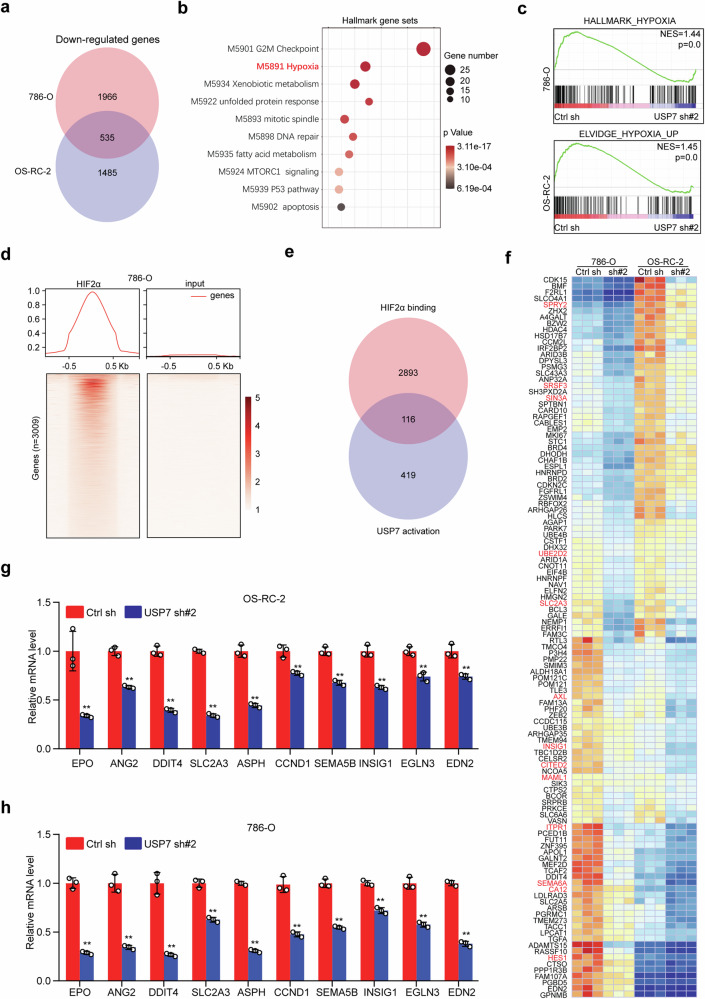
*USP7* knockdown impairs the HIF2α transcriptional program. **a** Venn diagram of downregulated genes resulting from USP7 knockdown in OS-RC-2 and 786-O cells. Downregulated genes were identified as those with *p* < 0.05, and FPKM > 1 using edgeR software. **b** Pathway enrichment analysis of 535 overlapped genes using the Hallmark Gene Sets database (https://metascape.org). **c** Gene set enrichment analysis (GSEA) of hypoxia-induced gene sets in the differential expression profile of 786-O and OS-RC-2 cells upon USP7 depletion (https://www.gsea-msigdb.org/gsea/index.jsp). **d** HIF2α ChIP-seq signal in 786-O is plotted for the promoters (TSS ± 1 kb, *n* = 3009) of the genes shown, where red indicates higher enrichment, GSE34871. **e** Venn diagram of 3009 HIF2α binding genes and 535 USP7-activated genes. **f** Heatmap presentation of 116 overlapped genes as shown in (**e**), the published HIF2α targets were marked in red. **g** and **h**. Downstream genes of HIF2α were analyzed by qRT-PCR in OS-RC-2 (**g**) and 786-O (**h**) cells with or without USP7 depletion. Graph shows mean ± SD from triplicates (**g** and **h**). **p < 0.01.

To explore the molecular mechanisms of USP7-mediated cell survival, RNA sequencing was performed using 786-O and OS-RC-2 cells with or without USP7 knockdown. Gene expression profiling revealed 2501 and 2020 downregulated genes in USP7-depleted 786-O and OS-RC-2 cells, respectively, of which 535 genes were overlapped (Fig. 3a). Pathway enrichment analysis using these 535 overlapped genes showed that USP7-associated pathways were involved in several important cellular processes, including cell cycle (G2M and mitotic spindle), cancer metabolism (xenobiotic and fatty acids) and ER stress (unfolded protein response). Surprisingly, the hypoxia pathway was significantly enriched, even though the samples were collected under normoxia (Fig. 3b). To confirm this enrichment, we respectively, interrogated the gene expression profile of 786-O and OS-RC-2 cells with previously validated hypoxia-induced gene signatures for gene set enrichment analysis (GSEA) [32], and revealed that USP7 knockdown markedly suppressed the expression of hypoxia-induced genes (Fig. 3c).

It has been well established that HIF2α, but not HIF1α, is a major oncogenic driver in ccRCC [10], as such, we reasoned that USP7 may regulate HIF2α downstream pathway. To verify this notion, HIF2α ChIP seq data in 786-O cells were analyzed (GSE34871), and 3009 genes were identified as potential HIF2α transcriptional targets (Fig. 3d). We interrogated these genes with the 535 USP7 activated genes, and revealed 116 overlapped genes, these included many previously identified canonical HIF2α targets including SLC2A3 [33], AXL [34] and INSIG1 [35] (Fig. 3e, f). Furthermore, we analyzed the HIF2α downstream gene expression in OS-RC-2 and 786-O cells by qRT-PCR, and consistently USP7 knockdown markedly suppressed these gene expressions (Fig. 3g, h). All these data argue that USP7 is required for HIF2α transcriptional activity.

### USP7 enhances HIF2α protein stability in vitro and in vivo

**Fig. 4:**
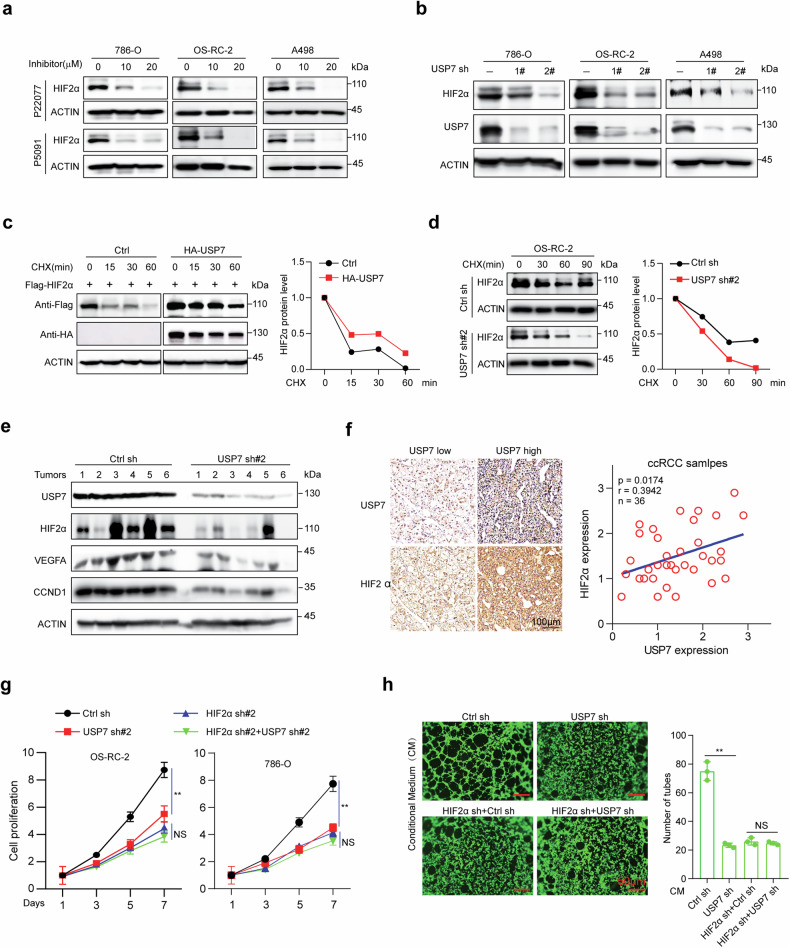
USP7 enhances HIF2α stability and oncogenic roles. **a** ccRCC cell lines were treated with the indicated concentration of P5091 or P22077 for 12 h, and the endogenous HIF2α protein level was analyzed by immunoblot using ACTIN as the loading control. **b** ccRCC cell lines were infected with control shRNA or USP7 shRNAs, and the endogenous USP7 and HIF2α protein levels were analyzed by immunoblot using ACTIN as the loading control. **c** 293T cells were transfected with indicated plasmids for 24 h and treated with cycloheximide (CHX, 100 μg/ml) for the indicated time, Flag-HIF2α level was analyzed and quantified by immunoblot with ACTIN as a loading control. **d** OS-RC-2 cells with or without USP7 knockdown were treated with CHX (100 μg/ml) for the indicated time, and endogeneous HIF2α level was analyzed and quantified by immunoblot with ACTIN as a loading control. **e** Immunoblots showing USP7, HIF2α, VEGFA, and CCND1 in xenograft tumors with or without USP7 knockdown by shRNA, six tumors were used per group, and ACTIN was used as a loading control. **f** IHC analysis of USP7 and HIF2α expression in a tissue microarray containing 36 ccRCC samples, representative images were shown (left) and the correlation between USP7 and HIF2α was analyzed (right). Scale bar, 100 μm. **g** Cells were infected with shRNAs targeting USP7 and/or HIF2α, and cell proliferation was assessed using CCK8. **h** Tube formation ability of HUVEC cultivated for 4 h in the conditional medium from infected OS-RC-2 cells, cells were treated with Calcein- AM. Scale bar, 50 μm. Pictures were taken with a fluorescence microscope, and tube numbers were counted. The experiments were independently repeated three times with similar results (**a**–**d**, **g** and **h**). Data are shown in mean ± SD. **p < 0.01, NS means no significant difference.

To explore whether USP7 influences HIF2α expression, 786-O, OS-RC-2, and A498 cells were treated with USP7 inhibitors P5091 or P22077, as shown in Fig. 4a, both inhibitors downregulated HIF2α protein expression in a dose-dependent manner. We next depleted USP7 by shRNAs in three ccRCC cell lines and found that USP7 knockdown significantly suppressed the HIF2α protein level, but showed minimal effects on its mRNA level in all three cell lines (Fig. 4b and Supplementary Fig. S3a). Half-life analysis showed that enforced expression of USP7 extended the half-life of HIF2α upon overexpression in 293T cells (Fig. 4c). Moreover, both enzymatic activity inhibition and knockdown of USP7 accelerated the degradation of endogenous HIF2α (Fig. 4d, Supplementary Fig. S3b). Furthermore, the 26S proteasome inhibitor MG132 significantly rescued the HIF2α loss caused by USP7 inhibition or knockdown (Supplementary Fig. S3c, d), arguing that USP7 depletion may promote proteasome-mediated HIF2α protein degradation.

We next measured the HIF2α abundance in xenograft tumors with or without USP7 depletion. As expected, USP7 inhibition or knockdown markedly depleted the intratumor HIF2α expression (Fig. 4e, Supplementary Fig. S3e). In addition, the representative HIF2α target genes, including vascular endothelial growth factor A (VEGFA) and cyclin D1 (CCND1) were also decreased (Fig. 4e, Supplementary Fig. S3e). Moreover, the intratumor microvascular density was analyzed by IHC staining of an endothelial cell marker CD31, and the results showed that USP7 depletion impaired angiogenesis within the tumor (Supplementary Fig. S3f, g). We further confirmed the USP7 and HIF2α expression in 36 ccRCC samples by IHC, and found that the expression of USP7 and HIF2α was significantly correlated (Fig. 4f). All these data demonstrate that the USP7–HIF2α axis may sustain HIF2α protein stability in vitro and in vivo.

To explore whether HIF2α is the major downstream effector of USP7, we depleted endogenous HIF2α expression by shRNAs before USP7 knockdown and revealed that USP7 knockdown showed minimal effects on cell proliferation in HIF2α-depleted cells (Fig. 4g, Supplementary Fig. S3h). Similarly, treatment of HUVEC cells with supernatant derived from USP7-silenced OS-RC-2 cells reduced the ability of tube formation, but depletion of USP7 did not affect the tube formation in HIF2α-deficient cells (Fig. 4h). Moreover, ectopic expression of HIF2α significantly rescued the proliferation inhibition in USP7-depleted cells (Supplementary Fig. S3i, j). These findings suggested that the oncogenic role of USP7 is mainly medicated by HIF2α.

### USP7 directly interacts with and deubiquitinates HIF2α

**Fig. 5:**
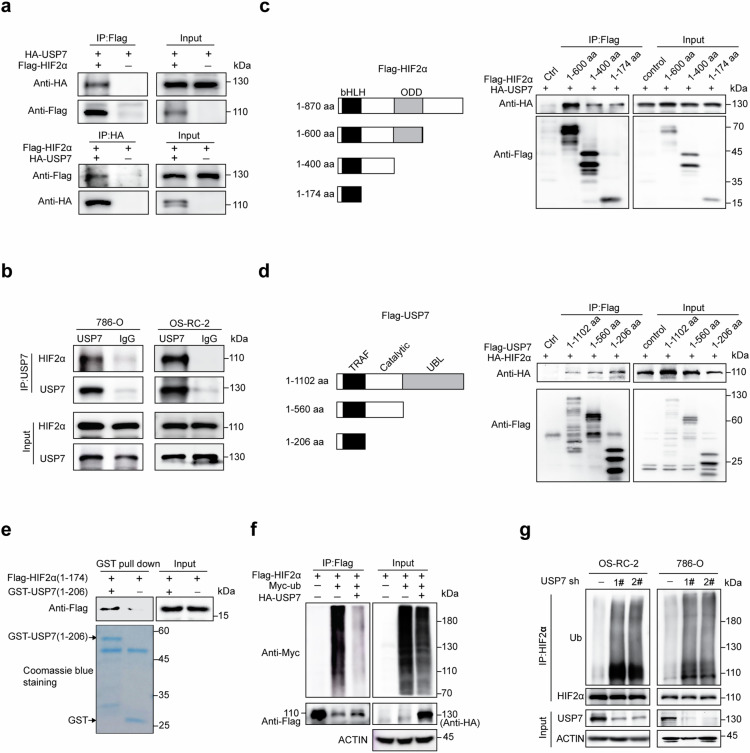
USP7 interacts with and deubiquitinates HIF2α. **a** 293T cells were co-transfected with Flag-HIF2α, HA-USP7, and/or the indicated vectors, the cell lysates were immunoprecipitated using anti-Flag or anti-HA antibodies and analyzed by immunoblotting with the indicated antibodies. **b** Lysates from OS-RC-2 or 786-O cells were subjected to immunoprecipitation using antibodies against USP7, and HIF2α protein was detected by immunoblots. **c** HEK293T cells were transfected with plasmids expressing Flag-HIF2α truncations and HA-USP7. The cell lysates were immunoprecipitated with an anti-Flag antibody and analyzed by immunoblotting with the indicated antibodies. **d** 293T cells were transfected with plasmids expressing Flag-USP7 or its truncations with HA-HIF2α, the cell lysates were immunoprecipitated with an anti-Flag antibody and analyzed by immunoblotting with the indicated antibodies. **e** GST pull-down assays were performed with the indicated GST-USP7 (1–206 aa) and cell lysates from 293T cells expressing Flag-HIF2α (1-174 aa), the immunoprecipitated Flag- HIF2 (1–174 aa) was analyzed by immunoblot. **f** 293T cells were co-transfected with the indicated plasmids. After 24 h, the cells were treated with 20 μM MG132 for 6 h and then subjected to denaturing immunoprecipitation using an anti-HA antibody followed by immunoblot analysis using the indicated antibodies. **g** ccRCC cells with or without USP7 depletion were treated with 20 μM MG13, 6 h later, cells were subjected to denaturing immunoprecipitation using HIF2α antibodies followed by immunoblot analysis using the indicated antibodies. The experiments were independently repeated three times with similar results (**a**–**g**).

To validate the physical interaction between USP7 and HIF2α, we overexpressed HA-USP7 and Flag-HIF2α in 293T cells for reciprocal immunoprecipitation and confirmed associations between both proteins (Fig. 5a). Moreover, co-immunoprecipitation using 786-O and OS-RC-2 cell lysates validated the specific interaction between endogenous USP7 and HIF2α in ccRCC cells (Fig. 5b). To define the minimal domain(s) in HIF2α for this interaction, we expressed full-length HA-tagged USP7 and respective Flag-tagged fragments of HIF2α in 293T cells, and the N terminal of HIF2 (1–174 aa) exhibits significant interaction with USP7 (Fig. 5c). Similarly, the N terminal of USP7 (1–206 aa) was also identified to bind with HIF2α upon overexpression in 293T cells (Fig. 5d). To evaluate the direct association between USP7 and HIF2α, we performed GST-pulldown assay with recombinant GST-tagged USP7 (1–206 aa) and eukaryotic Flag-tagged HIF2α (1–174 aa), and confirmed the direct association of these two regions (Fig. 5e).

We further assessed the influence of USP7 on the HIF2α polyubiquitination level. We co-transfected Flag-HIF2α, Myc-ub, and HA-USP7 into 293T cells, and revealed that enforced USP7 expression markedly suppressed the ubiquitination level of HIF2α (Fig. 5f). Depletion of endogenous USP7 in OS-RC-2 and 786-O cells by shRNAs or inhibitors significantly enhanced the polyubiquitination level of HIF2α (Fig. 5g and Supplementary Fig. S3k). These findings showed that HIF2α is a bona fide substrate of deubiquitinase USP7.

### USP7 depletion synergizes with afatinib in vitro

**Fig. 6:**
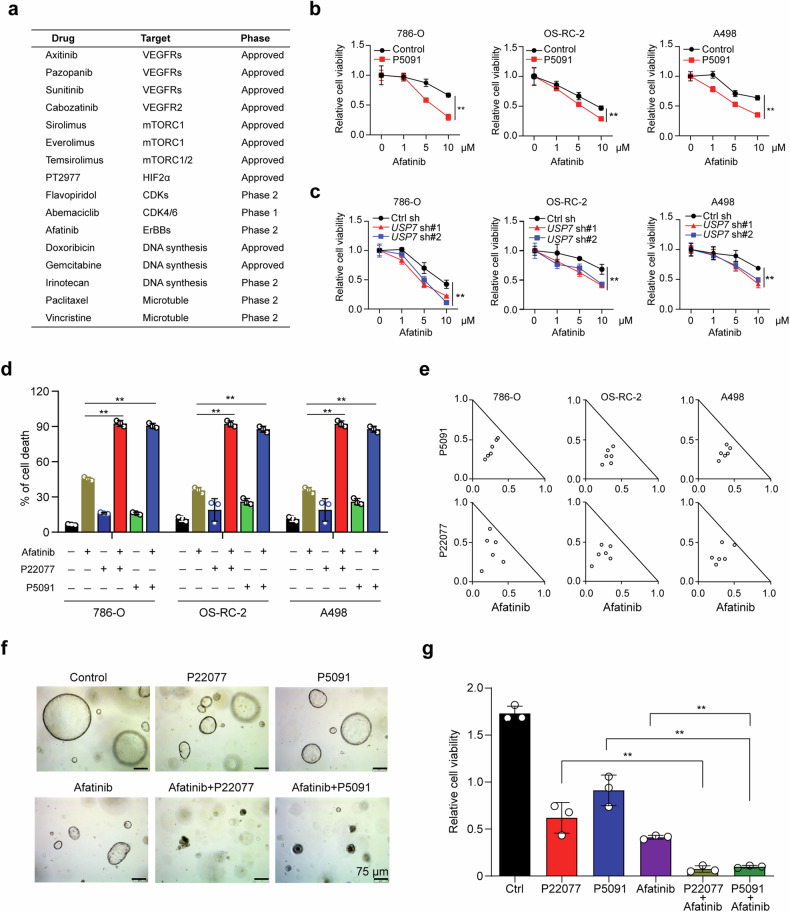
USP7 depletion synergizes with afatinib in *VHL* mutant ccRCC cells. **a** List of drugs used for combined drug screening. **b** Cells were seeded in 96-well plates with 1*10^4^ cells per well, 24 h later, cells were subjected to indicated concentration of afatinib with or without the combination of P5091 (10 μM) for 24 h, and cell viability was analyzed using CCK-8. **c** Cells with or without USP7 depletion were seeded in 96-well plates with 1*10^4^ cells per well, 24 h later, cells were treated with the indicated concentration of afatinib for another 24 h and then cell viability was measured using CCK-8. **d** ccRCC cells were treated with USP7 inhibitor and/or Afatinib for 48 h before cell death analysis (right) by Annexin V–PI staining. **e** Combination Index (CI) of P5091/P22077 and Afatinib was analyzed in ccRCC cells using the CompuSyn software (Biosoft). **f** ccRCC PDOs were seeded in 96-well plates, 48 h later, the PDOs were subjected to the single or combinational treatment of P22077 (1 μM), P5091 (1 μM), and Afatinib (1 μM) for 10 days, representative images of PDOs were shown. Scale bar, 75 μm. **g** PDO cell viability in (**f**) was measured by CellTiter-Glo® Luminescent Cell Viability Assay. The experiments were independently repeated three times with similar results and the graph shows mean ± SD from triplicates (**b**–**d** and **f**). **p < 0.01.

To improve the anti-tumor activity and durability of USP7 inhibitors, we performed a drug combination screen in OS-RC-2 cells from a collection of 16 drugs currently approved or in clinic trials for ccRCC patients and revealed that pharmacological inhibition of USP7 by P5091 significantly increased the sensitivity to afatinib and flavopiridol (Fig. 6a, b and Supplementary Fig. S4). P5091 treatment in another two cell lines also increased the sensitivity to afatinib, but not flavopiridol, indicating that the combination of USP7 inhibitor and afatinib is more global (Fig. 6b and Supplementary Fig. S5a). Afatinib is an irreversible inhibitor of the ERBB receptor family (including EGFR, HER2, ERBB3, and ERBB4), and has been approved for the treatment of advanced lung adenocarcinoma [22]. We further depleted USP7 in OS-RC-2 and 786-O cells by shRNA or another USP7 inhibitor P22077 and confirmed that USP7 depletion sensitizes ccRCC cells to afatinib (Fig. 6c and Supplementary Fig. S5b). Similarly, crystal violet staining and cell apoptosis assays showed that the combination of USP7 inhibitors and afatinib caused more profound cytotoxicity (Fig. 6d and Supplementary Fig. S5c, d), and the drug combination indices (CI) were <1, suggesting the synergistic lethal effect of the co-inhibition of USP7 and ERBB receptor (Fig. 6e). We also exploited the PDO for afatinib treatment with or without the combination of USP7 inhibitor P5091/P22077. As expected, treatment of afatinib alone suppressed organoid growth, and the combination of afatinib and P5091/P22077 led to a more pronounced inhibition (Fig. 6f, g).

### P5091 and afatinib synergistically suppress tumor progression in vivo

**Fig. 7:**
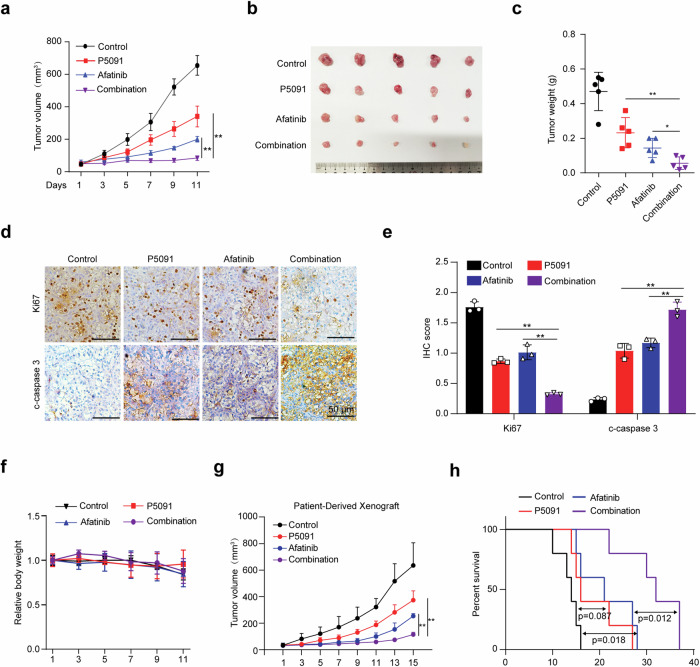
P5091 and afatinib synergistically suppress tumor progression in vivo. **a–c** Xenograft tumor growth (**a**), tumor image (**b**) and tumor weight (**c**) of OS-RC-2 cells after 11 days of treatment with saline, P5091 (IP, 25 mg/kg), afatinib (IG, 20 mg/kg), and P5091/afatinib combination. *n* = 5 for each group. **d** and **e** Representative histological images of Ki67 and cleaved caspase 3 staining in tumors with different treatments (**d**) and the histological stain was quantified using ImageJ and plotted in (**e**). Scale bar, 50 μm. **f** Relative mouse body weight during treatment. **g** PDXs tumor growth with the treatment of saline, P5091 (IP, 25 mg/kg), afatinib (IG, 20 mg/kg), and P5091/afatinib combination, *n* = 5 for each group. **h** Kaplan–Meier survival curve of mice in (**g**). Data are shown in mean ± SD, **p < 0.01.

To examine the consequences of dual inhibition of USP7 and ERBB receptor in vivo, we next used OS-RC-2 xenograft for dual treatment using P5091 and afatinib. As expected, treatment of P5091 or afatinib alone suppressed tumor growth, and their combination led to a more profound inhibition (Fig. 7b, c). IHC staining confirmed that the co-treatment showed the most reduced marker of proliferation Ki-67 and enhanced cleaved caspase-3 staining (Fig. 7d, e), arguing a more significant inhibition of tumor cell proliferation and intratumoral cell apoptosis in the xenograft tumors. Notably, the drug combination showed tolerable side effects, leading to minimal changes in mice body weight (Fig. 7f). We further explored the therapeutic efficacy of P5091 and/or afatinib in patient-derived xenografts (PDXs) derived from primary *VHL*-mutant ccRCC cells (patient in Fig. 2g). Treatment started when the tumors reached about 50 mm^3^ (at day 22 after implantation), and the tumor size was measured every other day. As shown in Fig. 7g, h, the combination of P5091 and afatinib synergistically inhibited ccRCC tumor growth and significantly extended the survival of recipient mice in comparison to the single treatment. As such, this combinational strategy holds great translational potential for ccRCC therapy.

### Afatinib destabilizes HIF2α by blocking FUBP1–USP7 signaling pathway

**Fig. 8:**
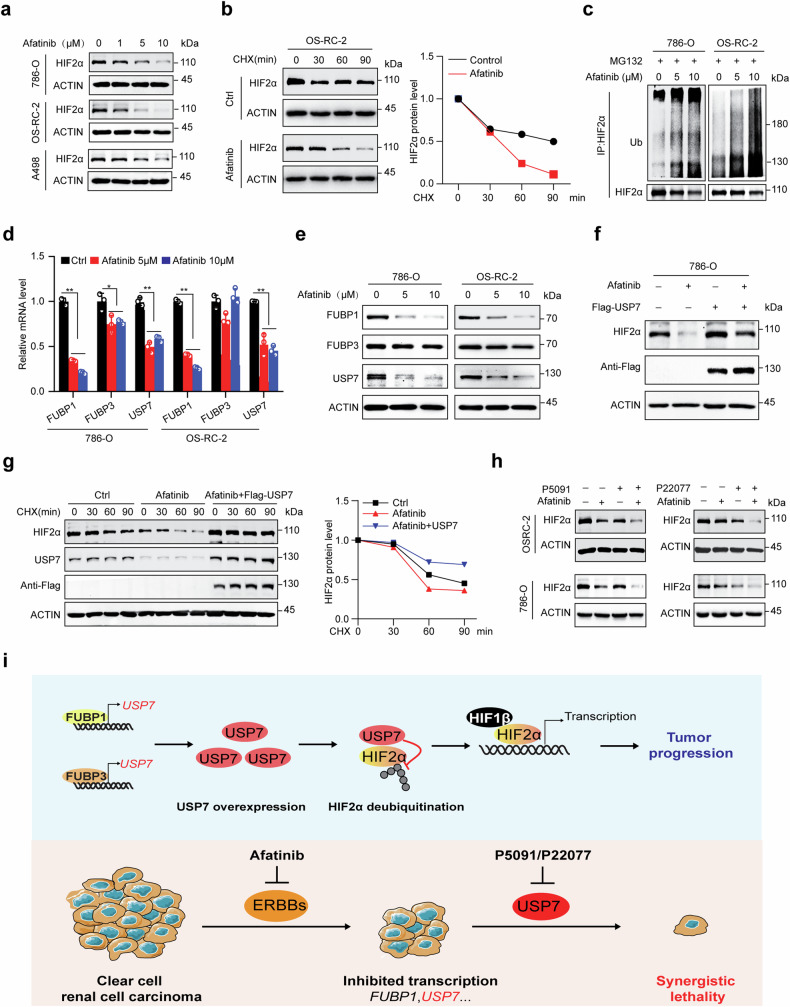
Afatinib degrades HIF2α by suppressing the FUBP1–USP7 regulatory axis. **a** Cells were treated with the indicated concentration of Afatinib for 24 h, and HIF2α protein levels were analyzed by immunoblots with ACTIN as loading control. **b** Cells with or without the pretreatment of 5 μM Afatinib for 24 h were subjected to the administration of CHX (100 μg/ml) for the indicated time, and HIF2α levels were analyzed and quantified with ACTIN as control. **c** Cells were pretreated with the indicated concentration of Afatinib for 24 h, and then treated with 20 μM MG132 for 6 h, the polyubiquitination level of HIF2α was analyzed by denaturing immunoprecipitation and immunoblots. **d** and **e** Cells were treated with the indicated concentration of Afatinib for 24 h, and FUBP1, FUBP3, and USP7 expression levels were analyzed by qRT-PCR (**d**) and immunoblots (**e**). **f** 786-O cells with or without Flag-USP7 expression were treated with 10 μM Afatinib for 24 h, and protein expression was analyzed using immunoblots. **g** 786-O cells with or without USP7 expression were pretreated with 5 μM Afatinib for 24 h, and then subjected to the administration of CHX (100 μg/ml) for the indicated time, HIF2α levels were analyzed and quantified with ACTIN as control. **h** Single or combinational treatment of P5091 (5 μM), P22077 (5 μM), and Afatinib (5 μM) for 24 h, HIF2α protein levels were analyzed by immunoblots with ACTIN as loading control. **i** Proposed a mechanism for the action of FUBP1/3-USP7-HIF2α regulatory axis in ccRCC tumor progression and the mechanism-based targeted strategy. The experiments were independently repeated three times with similar results (**a**–**h**), and the Graph shows mean ± SD from triplicates in one experiment (**d**). **p < 0.01, *p < 0.05.

We next explored the underlying mechanism of the drug combination. Interestingly, we found afatinib treatment decreased HIF2α protein abundance in a dose-dependent manner, while its’ mRNA level was increased (Fig. 8a and Supplementary Fig. S6a), indicating that afatinib may affect HIF2α expression at the protein level. Indeed, afatinib treatment markedly accelerated the degradation of endogenous HIF2α in 786-O and OS-RC-2 cells (Fig. 8b and Supplementary Fig. S6b). Afatinib increased the polyubiquitination level of HIF2α, and administration of proteasome inhibitor MG132 completely rescued afatinib-induced HIF2α loss (Fig. 8c and Supplementary Fig. S6c), arguing that afatinib promotes polyubiquitination medicated HIF2α degradation.

We further revealed that afatinib suppressed the gene transcription of FUBP1 and USP7 (Fig. 8d, e), indicating that afatinib may regulate HIF2α protein stability by suppressing the FUBP1–USP7 regulatory axis. As support, enforced expression of USP7 markedly rescued the abundance and half-life of HIF2α protein (Fig. 8f, g), as well as improved the cell resistance to afatinib (Supplementary Fig. S6d). Importantly, the combination of USP7 inhibitor P22077/P5091 and afatinib caused a more significant decrease in HIF2α protein expression compared to a single treatment (Fig. 8h). These findings indicated that dual inhibition of USP7 and ERBB receptor induces cell death partially by combinational degrading of HIF2α.

## Discussion

In this study, we have identified a novel FUBPs–USP7–HIF2α regulatory axis that modulates the progression of ccRCC, and developed a potential therapeutic strategy of ccRCC by combinational usage of USP7 inhibitor and afatinib (Fig. 8i). A previous study indicated that USP7 might interact with and deubiquitinate tumor suppressor ARMC5 in renal cell carcinoma, and silencing of USP7 promoted 786-O cell proliferation in vitro [36]. Nevertheless, the role of USP7 in ccRCC remains unknown. In our study, we found that depletion of USP7 by shRNAs or inhibitors caused significant suppression of cell proliferation in ccRCC cell lines, xenografts, PDO, mini-PDX, and PDX models, revealing a novel oncogenic role of USP7 in ccRCC. USP7 depletion was reported to suppress various tumor progressions by degradation of oncogenic E3 ligase MDM2, which leads to the re-activation of the tumor suppressor p53 [37]. In ccRCC, p53 was reported to be rarely mutated and functionally inhibited, and p53 status does not dictate HIF2α dependence in preclinical models [38, 39], indicating that USP7 depletion may suppress ccRCC cell proliferation independent of the MDM2-p53 pathway. In support of this notion, we revealed that USP7 could stabilize HIF2α, which is a key oncogenic driver in ccRCC [10]. In summary, we first uncovered the oncogenic role of USP7 and the key underlying mechanism in ccRCC.

HIF2α accumulation in ccRCCs has been thought to be driven by *VHL* gene deficiency [40], while there were some VHL-independent mechanisms exist [41–43]. Through a screen of deubiquitinase complementary DNA (cDNA) library that contains 60 deubiquitinases without USP7, Qing Zhang’s group identified USP37 as a HIF2α binding partner, which can deubiquitinate and stabilize HIF2α in ccRCC [43]. Our study identified USP7 as a novel HIF2α deubiquitinase, which was critical to its protein stability. Either USP37 or USP7 depletion caused a remarkable suppression of HIF2α protein levels and growth defect in VHL-deficient ccRCC cells, suggesting that deubiquitinating regulation plays a key role in maintaining HIF2α accumulation in ccRCCs.

Although HIF2α inhibition would be a powerful approach for the treatment of ccRCC, the current strategies were only designed to inhibit its transcriptional activity, which has a significant limitation [15–17]. Direct targeting of HIF2α for degradation has been a challenge for a long time owing to its “undruggable” protein structure, hence, the indirect strategy for degrading HIF2α may be an alternative. In this study, we developed a potential indirect HIF2α targeting strategy by USP7 inhibition. While the detailed mechanisms remain unclear, functional blocking of ERBB receptors by afatinib, which is in phase II clinical trial for renal cell cancers, was shown to induce proteasome-medicated HIF2α degradation by transcriptionally inhibiting USP7. These findings indicated that the ERBB signaling pathway may also contribute to oncogenic HIF2α accumulation in ccRCC, and ERBB receptor inhibitor afatinib may exhibit a more significant therapeutic effect on ccRCC, which may be helpful for patient selection in clinical trials. Moreover, we revealed that the combination of USP7 enzymatic inhibitor and afatinib caused a more profound inhibition of tumor progression in vitro and in vivo, partly by suppressing the USP7-HIF2α regulatory axis. Other mechanisms underlying the drug combination may also exist. In non-small cell lung cancer (NSCLC), USP7 was recently reported to deubiquitinate and stabilize estrogen receptor β (ERβ), and USP7-promoted ERβ stabilization is potentiated by ROS-induced stimulation. Moreover, depletion of USP7 induces ROS accumulation and overcomes the resistance to osimertinib, which is a first-line therapy in advanced NSCLC patients harboring EGFR-activating or T790 M resistance mutations [44]. These findings suggest that the co-inhibition of USP7 and the ERBB receptor family may represent a promising therapeutic strategy for cancer.

## Materials and methods

### Cell culture

ccRCC cell lines A498, OS-RC-2, 786-O, and Caki-2 were purchased from ProCell Life Science & Technology Company (Wuhan, China), HEK-293T was purchased from American Type Culture Collection (ATCC). Caki-2, A498, and 293T cells were maintained in DMEM (Hyclone, USA) medium containing 10% fetal bovine serum (FBS, Zeta Life, USA) and 1% penicillin/streptomycin (Hyclone, USA). OS-RC-2 and 786-O cells were grown in RPMI-1640 medium supplemented with 10% FBS and 1% penicillin/streptomycin. HUVEC cells were purchased from SAIOS company (CL-191h, Wuhan, China) and cultured in an endothelial cell medium (Sciencell, cat#1001). All cells were cultured at 37 °C with 5% CO_2_.

### Antibodies and reagents

Rabbit anti-USP7 (A3448, A13564), rabbit anti-Flag tag (AE004), mouse anti-HA tag (AE008), rabbit anti-Ubiquitin (A0162), rabbit anti-Ki67 (A20018), and rabbit anti-Caspase-3 (A11021) were obtained from ABclonal (Wuhan, China). Rabbit anti-HIF2α (ab109616) was obtained from Abcam. Rabbit anti-VEGF-A (65373) and rabbit anti-CCND1 (55506) were obtained from Cell Signaling Technology. Rabbit anti-CD31(28083-1-AP) was purchased from Proteintech (Wuhan, China). MG132 (T2154) and afatinib (T21312) were obtained from Topscience (Shanghai, China). Cycloheximide (HY-12320), P5091 (HY-15667), and P22077 (HY-13865) were obtained from MedChemExpress. Trizol reagent was obtained from Sangon Biotech (Shanghai, China). The tissue microarrays of human ccRCC (U081Ki01) were purchased from Bioaitech (Xian, China).

### Tissue processing

The ccRCC samples were obtained from patients who had not received chemotherapy or radiotherapy before the operation. The protocols for human studies were approved by the Institute Research Medical Ethics Committee of The First Affiliated Hospital of Xi’an Jiaotong University, and the patients signed the consent form. The tumor tissues were collected during surgery and stored in the DMEM medium (containing 2× penicillin–streptomycin) for transportation. The tissues were cut into 3–5 mm^3^ pieces and randomly divided into three parts. One part was snap frozen and stored at −80 °C for sequencing, one part was fixed in 10% formalin for histopathological analysis and immunohistochemistry, and the last part was used for establishing PDO, Mini-PDX, and PDX models.

### Organoid culture and passaging

The primary cell pellet was suspended with 10 μL DMEM/F12 (Gibco, USA) and 50 μL GFR Matrigel (Corning, USA), and solidified in the 24-well plate (Corning, USA) at 37 °C for 30 min, 500 μL organoid medium was added to the well and the plate was transferred to 37 °C/5% CO_2_ incubator for culture. The organoid medium contains DMEM/F12 supplemented with B27 supplement (1.5×, Gibco, USA), FGF10 (100 ng/mL, Sino Biological, China), N-Acetylcysteine (0.625 mM, Sigma, USA), R-Spondin 1 (1 μg/mL, Sino Biological, China), Noggin (100 ng/mL, Sino Biological, China), A83-01 (2.5 μM, Abmole Bioscience, USA), EGF (500 ng/mL, Peprotech, USA), Y-27632 (10 μM), Primocin (100 μg/mL, Invitrogen, USA). Mediums were replaced every 5 days, and the organoids were passaged every 1–3 weeks. For organoids passaging, dense organoids were incubated with 1 mL TrypLE Express (Gibco, USA) at 37 °C for 10 min, suspended with 2 mL DMEM/F12, collected by centrifugation at 500 × *g*, and then seeded into a new plate.

### Animal studies

Mini-PDX models were established as described previously [45]. Briefly, the primary ccRCC cell suspension was transferred to Hank’s balanced salt solution (HBSS) washed capsules, and then embedded in the subcutaneous tissue of 6-week-old BALB/c-Nude mice (GemPharmatech, Nanjing, China). Drug P5091 (20 mg/kg, IP) or saline was administered for 7 days. The tumor cell viability was detected using the CellTiter-Glo® Luminescent Cell Viability Assay (Promega, Madison, WI, USA). For the xenograft model, 6 weeks-old female BALB/c nude mice (GemPharmatech, Nanjing, China) were injected subcutaneously with 5 × 10^6^ OS-RC-2 cells (with or without *USP7* knockdown) diluted in 100 μl PBS. For drug treatment, when tumors reached a volume of about 100 mm^3^, mice were randomly divided into four groups and subjected to the treatment of P5091 (25 mg/kg, IP), Afatinib (20 mg/kg, IG) alone or in combination once a day. Tumor volumes were measured since treatments began, and tumor weights were assessed in sacrificed animals.

For the PDXs model, human primary ccRCC tumors were transplanted into 6-week-old female NCG mice (GemPharmatech, Nanjing, China) four passages before the experiment. Tumor slices with an average volume of 5 mm^3^ were subcutaneously inoculated into recipients. when tumors reached a volume of about 50 mm^3^, mice were randomly divided into four groups and subjected to the treatment of P5091 (25 mg/kg, IP), afatinib (20 mg/kg, IG) alone or in combination once a day. Tumor volume was measured every other day, and mice were monitored for survival and euthanized when moribund or demonstrating obvious clinical distress.

Mice used for mini-PDX and xenograft models were maintained in the Animal Center of Xi’an Jiaotong University under specific pathogen-free (SPF) conditions. Mice used for PDX were maintained in the Animal Center of the First Affiliated Hospital of Nanchang University under specific pathogen-free (SPF) conditions. All animal experiments were performed with approval from the Institutional Animal Care and Use Committee.

### RNA sequencing

RNA sequencing was. OS-RC-2 and 786-O cells were infected with lentiviruses expressing control shRNA or USP7 shRNA, total RNA was extracted using TRIzol reagent (Thermo Fisher Scientific). RNA sequencing was performed by Shanghai Personal Biotechnology company. Significant differential expressed genes were identified as those with *p* < 0.05, and FPKM > 1 using edgeR software. The accession number for the gene expression data reported in this paper is BioProject database: PRJNA992200.

### RNA quantification

Total RNA was extracted with Trizol reagent (Thermo Fisher Scientific) following the manufacturer’s instructions. cDNA was reverse transcribed using HiScript II 1st Strand cDNA Synthesis Kit R211 (Vazyme, Nanjing, China). Quantitative PCR was conducted using 2 × RealStar Fast SYBR qPCR Mix (Genstar, Beijing, China). Relative expression of the mRNA was calculated by the 2^−ΔΔCt^ method and normalized to ACTIN using specific primers as shown in Supplementary Table S1.

### Cell viability assay

Cells were seeded into 96-well plates (1*10^4^ cells per well) and cultured for 24 h, then treated with an increasing dosage of drugs for 24 h, respectively. The cell viability was assayed using Cell Counting Kit-8 according to the manufacturer’s instructions. For the cell proliferation assay, cells were seeded into 96-well plates at a density of 1*10^3^ cells per well, and the cell viability was determined at the indicated time. For Organoids, the cell viability was determined using the CellTiter-Glo® Luminescent Cell Viability Assay (Promega, Madison, WI, USA).

### Tubule formation

OS-RC-2 cells infected with different shRNAs were counted and seeded into the six-well plate, 24 h later, the medium was collected as the conditional medium. The tubule formation assay was performed as described [46]. HUEVC cells were digested and washed three times using PBS and then resuspended with different conditional medium. The 96-well plate was coated with 70 µl matrigel per well, and cells were seeded in the matrigel-coated 96-well plate with a density of 3 × 10^4^ cells per well. After 4 h, cells were stained with Calcein AM (Beyotime Biotechnology, C2013S), pictures were taken with a fluorescence microscope, and the tube number was counted.

### Ubiquitination analysis

Cells were collected with PBS and lysed in 100 μL SDS lysis buffer (50 mM Tris–HCl, 150 mM NaCl, 1% NP-40, 1% SDS, 1 mM EDTA, pH 7.4). The lysate was sonicated, denatured at 95 °C for 15 min to disrupt protein interaction, and then diluted with 900 μL NP40 buffer (50 mM Tris–HCl, 150 mM NaCl, 1% NP-40, 1 mM EDTA, pH 7.4). The cell lysates were subjected to centrifugation at 12,000×*g* for 15 min. 10% of supernatant was saved as input to detect protein expression, and the remaining cell extract underwent immunoprecipitation with specific antibodies, followed by immunoblot analysis of protein polyubiquitination.

### Immunohistochemistry

Immunohistochemistry(IHC) was performed using the immunohistochemistry kit (proteintech, PK10006). The tissue microarrays of human renal cancer were purchased from Bioaitech (U081Ki01, Xi’an, China). The IHC scores were measured as previously published [47, 48]. In brief, each sample was scored based on the staining intensity: negative staining was 0, weak positive was 1, positive was 2, and strong positive was 3.

### Statistical analysis

Data analysis was carried out using GraphPad Prism 8. Statistical significance between two groups was calculated by unpaired two-tailed Student’s *t*-test, when comparing three or more groups, one-way or two-way ANOVA with Tukey’s corrections was used. Differences were considered significant when *p* < 0.05.

## Supplementary information

Supplementary Materials

Raw data for Western Blot experiment

## Data Availability

All data are available in the main text or the supplementary information. The accession number for the gene expression data reported in this manuscript is BioProject database: PRJNA992200.
