# Supplementary material for: USP7 depletion potentiates HIF2α degradation and inhibits clear cell renal cell carcinoma progression
Source: Cell Death Dis. 2024 Oct 15;15(10):749. doi: 10.1038/s41419-024-07136-0 (PMC11482519; doi:10.1038/s41419-024-07136-0)
Supplement: Supplementary file 1 — Supplementary Materials [file 41419_2024_7136_MOESM1_ESM.pdf]

Supplementary Information

USP7 depletion potentiates HIF2α degradation and inhibits clear cell renal cell carcinoma progression

Rongfu Tu, Junpeng Ma, Yule Chen, Ye Kang, Doudou Ren, Zeqiong Cai, Ru Zhang, Yiwen Pan, Yijia Liu, Yanyan Da, Yao Xu, Yahuan Yu, Donghai Wang, Jingchao Wang, Xinlan Lu, Chengsheng Zhang

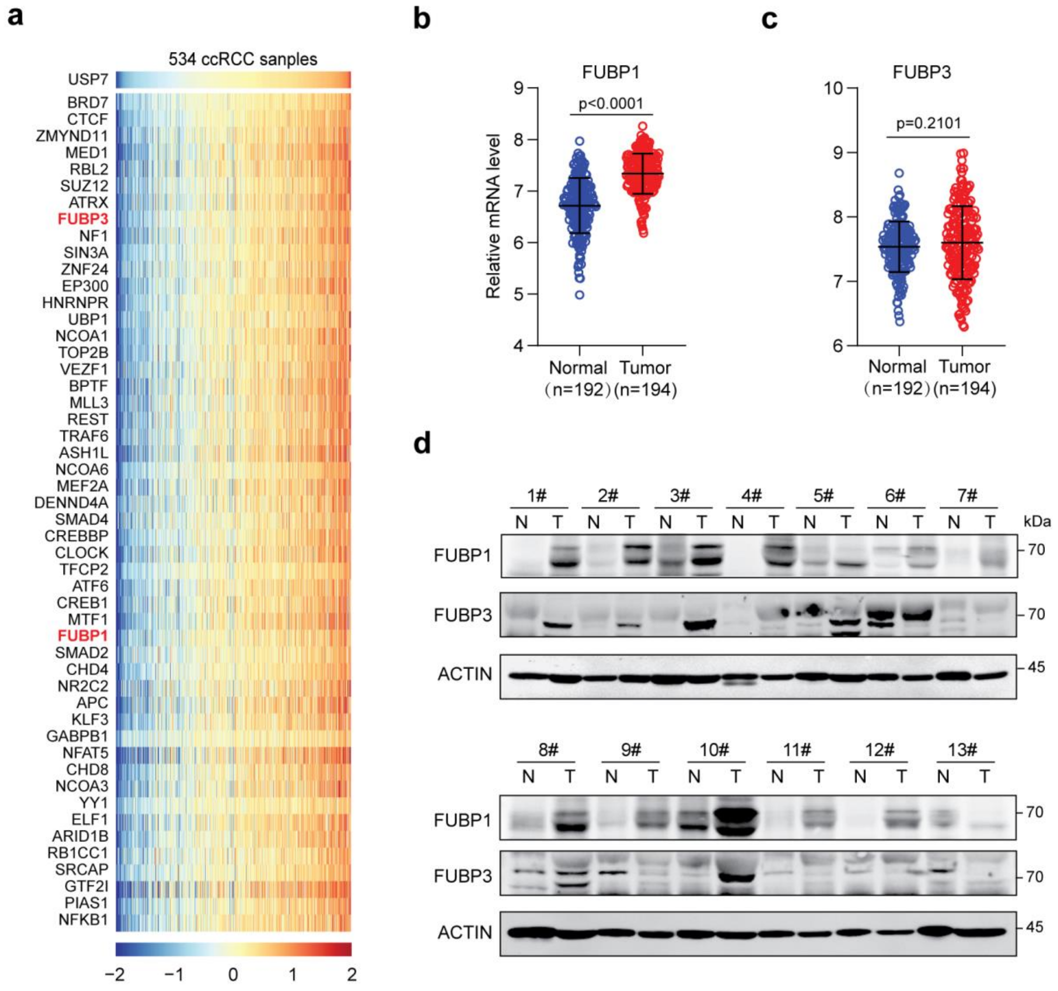

Supplementary Figure S1. Related to Figure1

a. Heatmap of top 50 transcription factors highly correlated with USP7 genes in 534 ccRCC samples. 795 human transcription factor list was obtained from the TRRUST database (https://www.grnpedia.org/trrust/), and the expression data was downloaded from the TCGA database (https://www.cancer.gov/ccg/research/genome-sequencing/tcga). **b** and **c**. Analysis of relative FUBP1(b) and FUBP3(c) mRNA level in normal kidney (Normal) and ccRCC (Tumor) tissues using R2 Genomics Analysis and Visualization Platform (https://hgserver1.amc.nl/). **d.** FUBP1 and FUBP3 protein expression in 13 ccRCCs and paired non-cancerous tissues were analyzed using Western blot.

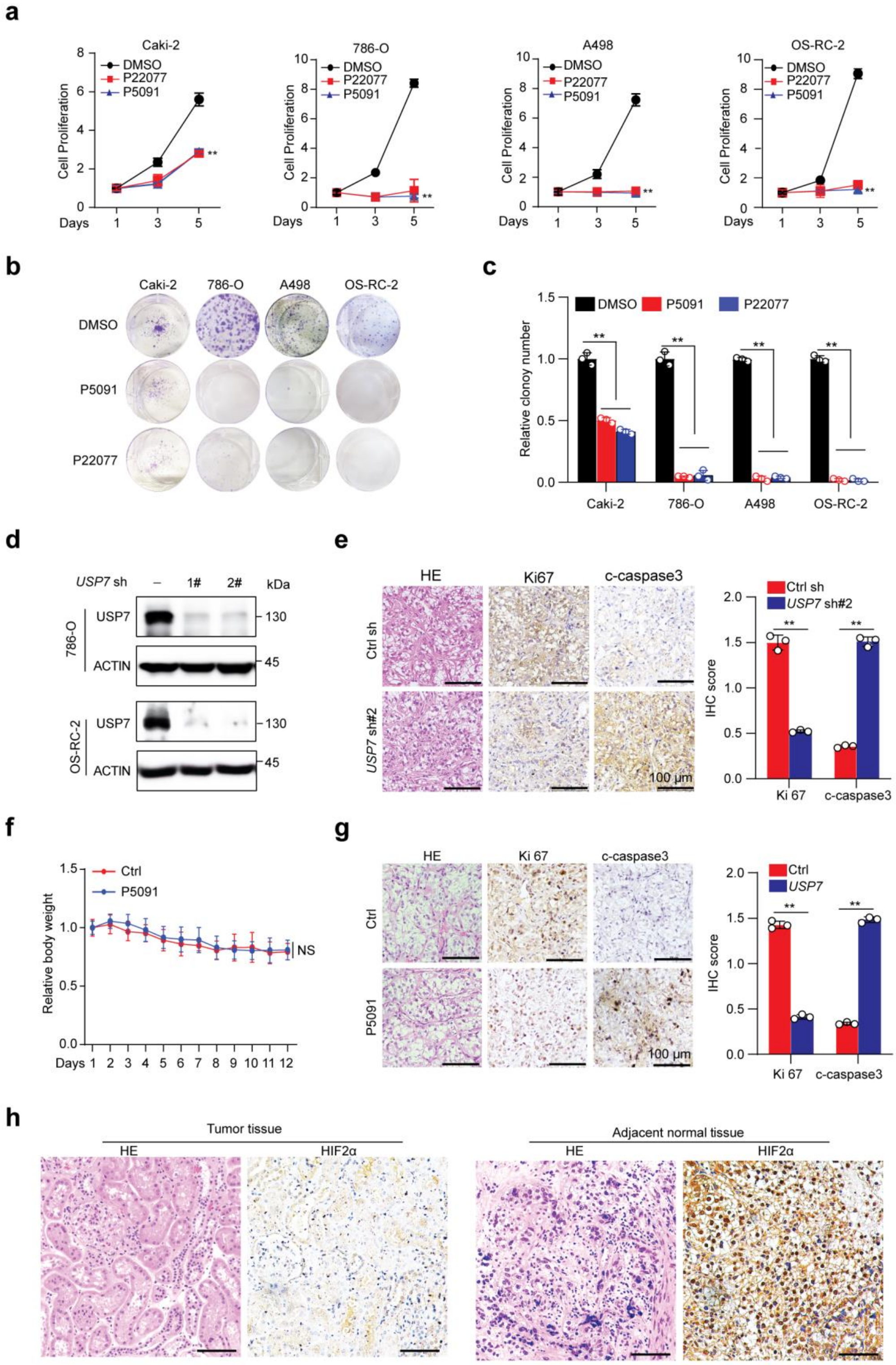
a
Caki-2
786-O
A498
OS-RC-2
DMSO
P22077
P5091
Cell Proliferation
Days
**
b
DMSO
P5091
P22077
c
Relative clony number
d
USP7 sh
1#
2#
kDa
USP7
ACTIN
130
45
e
HE
Ki67
c-caspase3
Ctrl sh
USP7 sh#2
100 μm
IHC score
Ki 67
f
Ctrl
Relative body weight
NS
g
USP7
h
Tumor tissue
Adjacent normal tissue
HIF2α

Supplementary Figure S2. Related to Figure 2

a. Proliferation of ccRCC cells with or without the treatment of 10 μM P5091 or 10 μM P22077. **b** and **c.** ccRCC cells were seeded into 6-well plates with 2000 cells per well, 24 h later, cells were treated with 5 μM P5091 or P22077, about 10 days later, the clones were stained with crystal violet (b), and the colony number was counted (c). **d.** 786-O and OS-RC-2 cells were infected with ctrl shRNA or USP7 shRNAs, and USP7 protein levels were analyzed using immunoblot. **e.** left: representative staining of HE, Ki67 and cleaved caspase 3 in control and USP7 depleted tumors, right: quantification of Ki67 and cleaved caspase-3. **f.** Relative body weight of mice with or without P5091 treatment (n=6). **g.** left: representative staining of HE, Ki67, and cleaved caspase 3 in control and P5091 treated tumors, right: quantification of Ki67 and cleaved caspase 3. **h.** Representative HE staining and IHC staining of HIF2α in renal cancer and adjacent normal tissues.

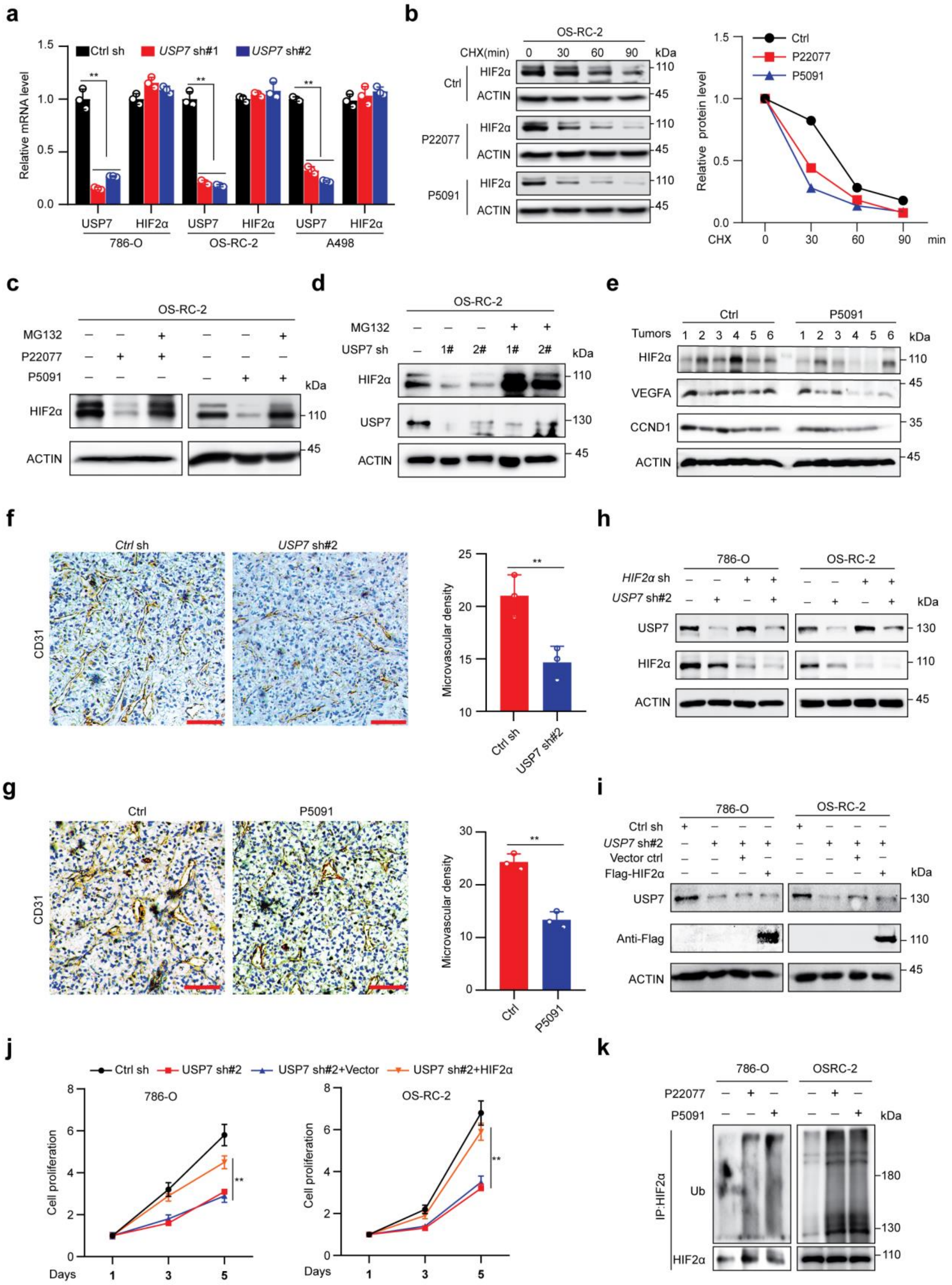
a
Ctrl sh
USP7 sh#1
USP7 sh#2
Relative mRNA level
1.5
1.0
0.5
0.0
**
USP7
HIF2α
786-O
OS-RC-2
A498
b
OS-RC-2
CHX(min)
0
30
60
90
kDa
Ctrl
P22077
P5091
HIF2α
ACTIN
110
45
Ctrl
P22077
P5091
Relative protein level
CHX
min
c
OS-RC-2
MG132
P22077
P5091
kDa
HIF2α
ACTIN
110
45
d
OS-RC-2
MG132
USP7 sh
1#
2#
kDa
HIF2α
USP7
ACTIN
110
130
45
e
Ctrl
P5091
Tumors
1
2
3
4
5
6
kDa
HIF2α
VEGFA
CCND1
ACTIN
110
45
35
f
Ctrl sh
USP7 sh#2
CD31
Microvascular density
25
20
15
10
**
g
Ctrl
P5091
CD31
Microvascular density
30
20
10
0
**
h
786-O
OS-RC-2
HIF2α sh
USP7 sh#2
kDa
USP7
HIF2α
ACTIN
130
110
45
i
786-O
OS-RC-2
Ctrl sh
USP7 sh#2
Vector ctrl
Flag-HIF2α
kDa
USP7
Anti-Flag
ACTIN
130
110
45
j
Ctrl sh
USP7 sh#2
USP7 sh#2+Vector
USP7 sh#2+HIF2α
786-O
OS-RC-2
Cell proliferation
8
6
4
2
0
Days
1
3
5
**
k
786-O
OSRC-2
P22077
P5091
kDa
IP:HIF2α
Ub
HIF2α
180
130
110

Supplementary Figure S3. Related to Figures 4 and 5.

a. ccRCC Cells were infected with control or USP7 shRNAs, and the mRNA levels of USP7 and HIF2α were analyzed by qRT-PCR. **b.** OS-RC-2 cells were treated with DMSO, P22077（10 μM）, or P5091（10 μM）for 12h before the treatment of CHX for the indicated time, HIF2α level was analyzed and quantified by immunoblot with ACTIN as a loading control. **c.** Immunoblots of HIF2α in OS-RC-2 cells in the presence of P5091, P22077, and/or MG132. Cells were treated with P5091 or P22077 (10 μM) for 12 h and subjected to MG132 (20 μM) for 6 hr before harvest as indicated.d. OS-RC-2 cells with or without USP7 depletion were treated with MG132 (20 μM) for 6 h before harvest, the HIF2α protein level were analyzed by immunoblots with ACTIN as loading control. **e.** Immunoblots showing HIF2α, VEGFA, and CCND1 in xenograft tumors with or without P5091 treatment , 6 tumors were used per group and ACTIN was used as a loading control. **f.** Tumor microvascular was analyzed by IHC analysis of CD31 expression in xenograft tumors with or without USP7 depletion, representative images were shown (left) and the microvascular number was conuted(right). **g.** Tumor microvascular was analyzed by IHC analysis of CD31 expression in xenograft tumors with or without P5091 treatment, representative images were shown (left) and the microvascular number was counted (right). **h.** Cells were infected with control or indicated shRNAs, USP7 and HIF2α protein expression was analyzed by immunoblots. **I** and **j.** Cells were infected with indicated plasmids, the protein expression was analyzed by immunoblots using indicated antibodies(i), and the cell proliferation was measured by CCK8(j). **k**. Cells were treated with P5091 or P22077 (10 μM) for 12 h and subjected to MG132 (20 μM) for 6 hr before harvest, the polyubiquitination levels of HIF2α were analyzed by denaturing immunoprecipitation and immunoblots.

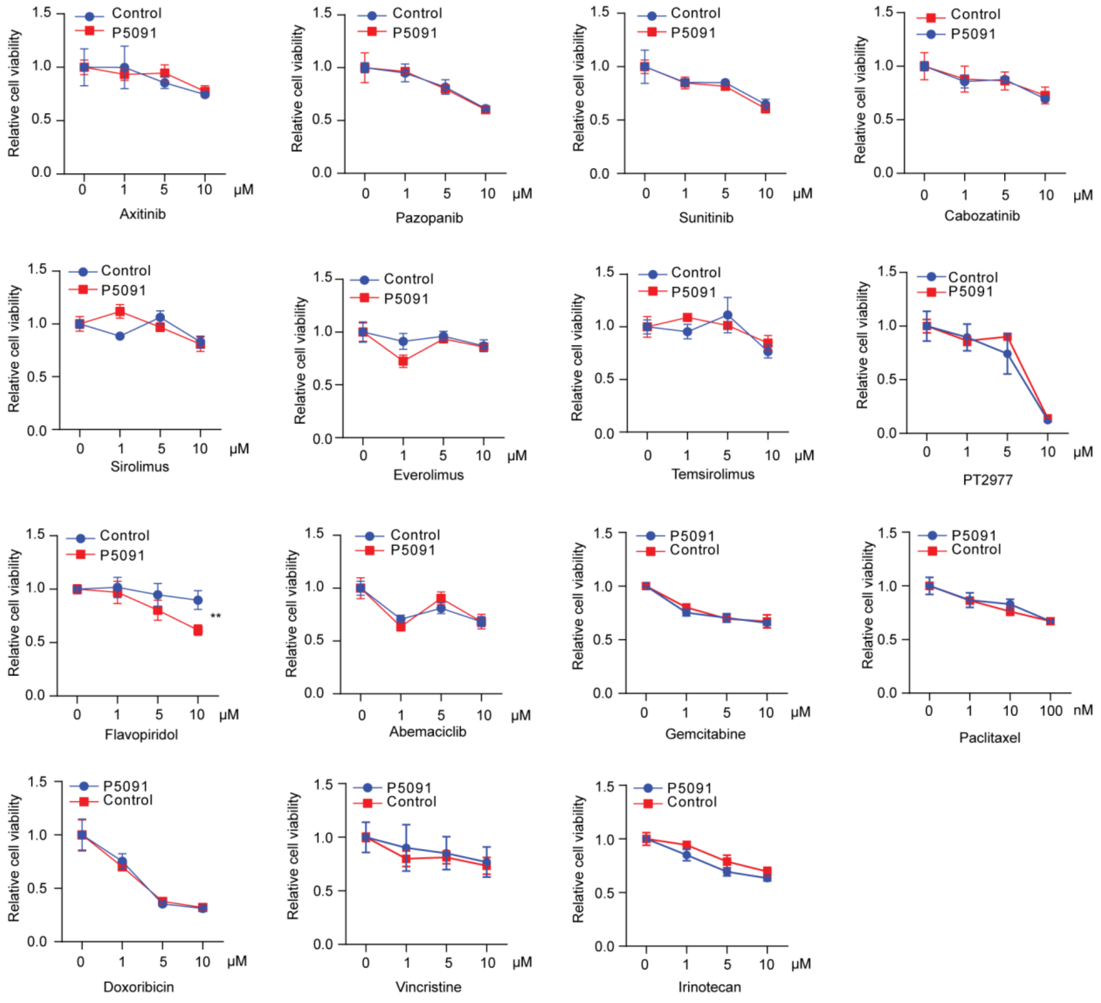

Supplementary Figure S4, related to Figure 6.

OS-RC-2 cells with or without the presentence of 10 μM P5091 were subjected to indicated concentration drugs for 24h. Cell viability was analyzed using CCK-8. The experiments were independently repeated three times with similar results, and the Graph shows mean ± SD from triplicates in one experiment

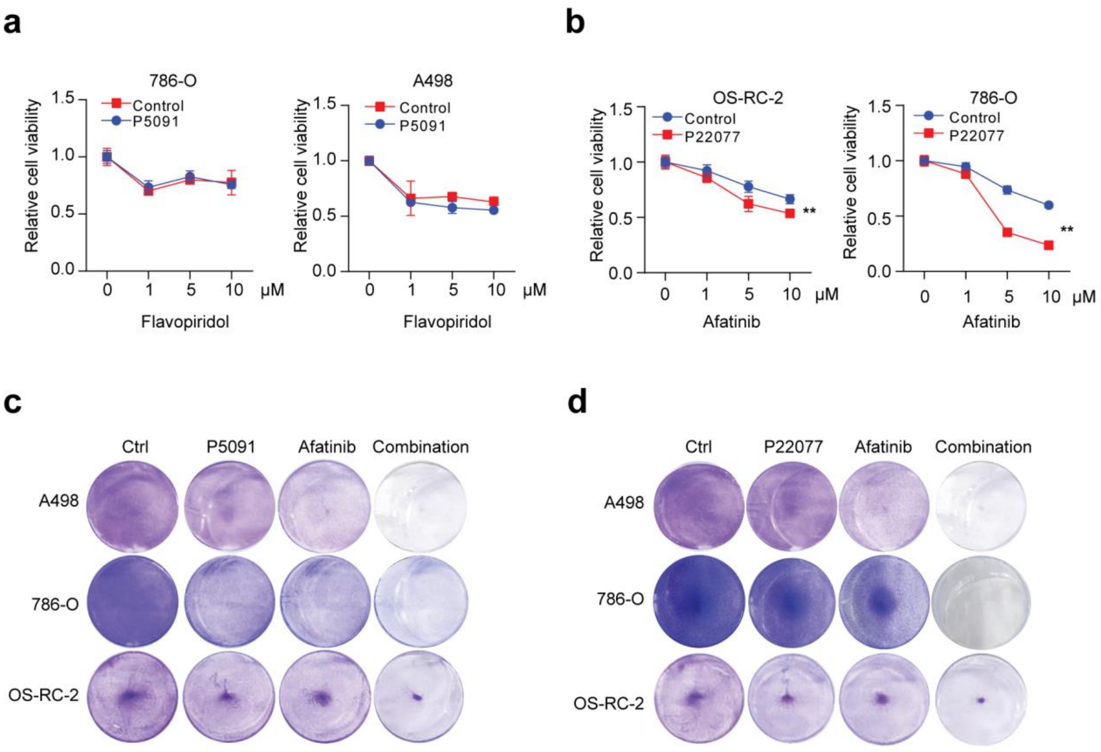

Supplementary Figure S5, related to Figure 6.

a. 786-O and A498 cells subjected to indicated concentration of Flavopiridol with or without the presentence of 10 μM P5091 for 24h, cell viability was analyzed using CCK-8. **b.** OR-RC-2 and 786-O cells subjected to the indicated concentration of afatinib with or without presentence of 10 μM P22077 for 24h, cell viability was analyzed using CCK-8. **c** and **d**. Cells were seeded in 6 well plates, 12 h later, the cells were treated with a single drug or combination of P5091 (10 μM)/P22077 and afatinib (5μM) for 48 h, the clones were stained with crystal violet and photographed. The experiments were independently repeated three times with similar results, and the Graph shows mean ± SD from triplicates in one experiment (a and b).

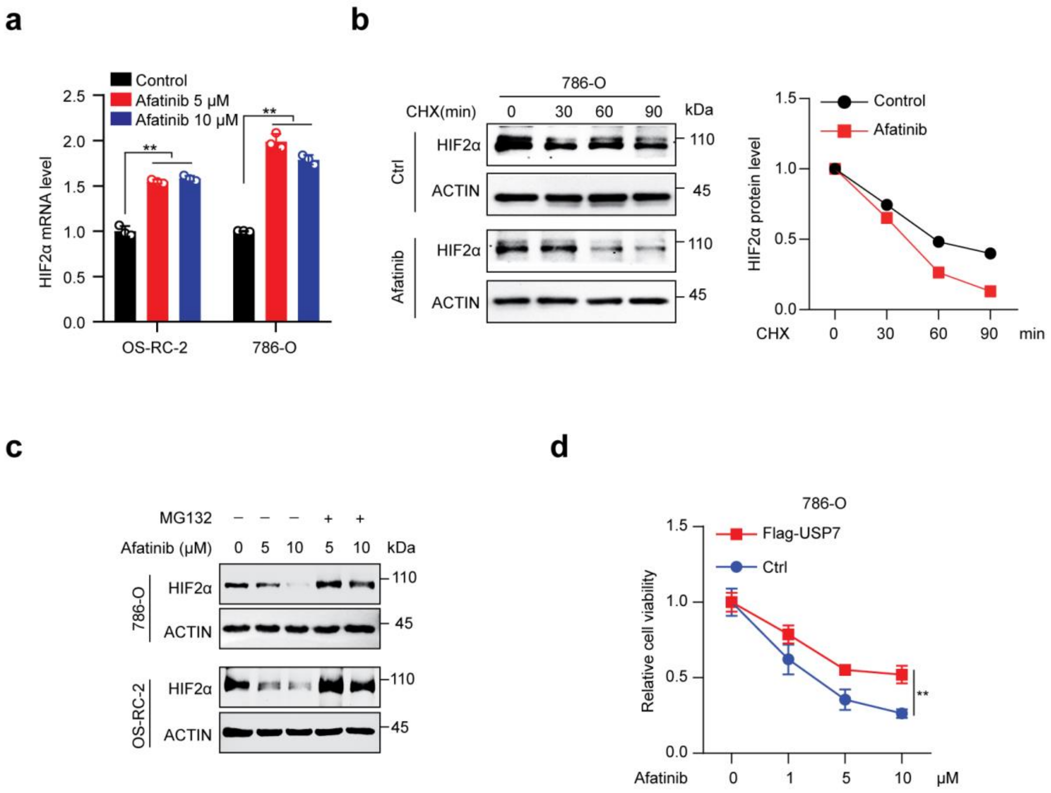

Supplementary Figure S6. Related to Figure 8.

a. Cells were treated with the indicated concentration of afatinib for 24 h, and the mRNA level of HIF2α was analyzed by qRT-PCR with ACTIN as a control. **b.** Cells were pretreated with the indicated concentration of afatinib for 24 h, and then treated with 20 μM MG132 for 6 h, HIF2α levels were analyzed by immunoblots with ACTIN as loading control. **c.** 786-O cells were treated with DMSO or Afatinib（5 μM）for 24h before the treatment of CHX for the indicated time, HIF2α level was analyzed and quantified by immunoblot with ACTIN as a loading control.**d**. Cells with or without overexpression of USP7 were treated with indicated concentration of afatinib for 24 h, and then cell viability was measured by CCK8.

Supplementary Table S1. Primers used in this study

Primers for qRT-PCR	
FUBP1 forward	5’-CAACCAGATGCTAAGAAAGTTGC-3’
FUBP1 reverse	5’-CCTCCTCTGCCAATTATGAATCC-3’
FUBP3 forward	5’-TCCGGCAGATTGCTGCTAAAA-3’
FUBP3 reverse	5’-CCGTATCCATATACTGAGGGGTC-3’
USP7 forward	5’-GGAAGCGGGAGATACAGATGA-3’
USP7 reverse	5’-AAGGACCGACTCACTCAGTCT-3’
HIF2α forward	5’-GGACTTACACAGGTGGAGCTA-3’
HIF2α reverse	5’-TCTCACGAATCTCCTCATGGT-3’
EPO forward	5’-GGAGGCCGAGAATATCACGAC-3’
EPO reverse	5’-CCCTGCCAGACTTCTACGG-3’
ANG2 forward	5’-AACTTTCGGAAGAGCATGGAC-3’
ANG2 reverse	5’-CGAGTCATCGTATTCGAGCGG-3’
DDIT4 forward	5’-TGAGGATGAACACTTGTGTGC-3’
DDIT4 reverse	5’-CCAACTGGCTAGGCATCAGC-3’
SLC2A3 forward	5’-GCTGGGCATCGTTGTTGGA-3’
SLC2A3 reverse	5’-GCACTTTGTAGGATAGCAGGAAG-5’
ASPH forward	5’-CATGGAGGACACAAGAATGGG-3’
ASPH reverse	5’-CCAAACGACAGCTACAGATGT-3’
CCND1 forward	5’-CAATGACCCCGCACGATTTC-3’
CCND1 reverse	5’-CATGGAGGGCGGATTGGAA-3’
SEMA5B forward	5’-TACGTGCGAGTCCTGATCGT-3’
SEMA5B reverse	5’-TCTTCTCAATAGTCCGGCTGA-3’
INSIG1 forward	5’-ATCCAGAGGAATGTCACTCTCTT-3’
INSIG1 reverse	5’-AGGGGTACAGTAGGCCAACAA-3’
EGLN3 forward	5’-CTGGGCAAATACTACGTCAAGG-3’
EGLN3 reverse	5’-CTGGGCAAATACTACGTCAAGG-3’
EDN2 forward	5’-CGTCCTCATCTCATGCCCAAG-3’
EDN2 reverse	5’-AGGCCGTAAGGAGCTGTCT-3’
ACTIN forward	5’-CATGTACGTTGCTATCCAGGC-3’
ACTIN reverse	5’-CTCCTTAATGTCACGCACGAT-3’
shRNA	
FUBP1 sh#1	5’-ACTACTGATAGGAGGTTAATA-3’
FUBP1 sh#2	5’-TACAACCCTGCACCTTATAAT-3’
FUBP3 sh#1	5’-CGGCGATTTCAACTCTCGAAT-3’
FUBP3 sh#2	5’-CCTGGCTTTCATAATGACATA-3’
USP7 sh#1	5’-CCTGGATTTGTGGTTACGTTA-3’
USP7 sh#2	5’-TGTATCTATTGACTGCCCTTT-3’
Ctrl shRNA	5’-TACAACAGCCACAACGTCTAT-3’
Primers for ChIP	

ACTIN promoter forward	5'-GACTTCTAAGTGGCCGCAAG-3'
ACTIN promoter reverse	5'-TTGCCGACTTCAGAGCAAC-3'
USP7 #1 forward	5’- CCAGGCTAGTCTCGAATTCCT -3’
USP7 #1 reverse	5’- AGAATCACTTGAACCCGGGA -3’
USP7 #2 forward	5’- CTGAGTAGCTGGGACCACA -3’
USP7 #2 reverse	5’- TGAGGTCAGGAGGTCGAGA -3’
