# Supplementary material for: USP7 depletion potentiates HIF2α degradation and inhibits clear cell renal cell carcinoma progression
Source: Cell Death Dis. 2024 Oct 15;15(10):749. doi: 10.1038/s41419-024-07136-0 (PMC11482519; doi:10.1038/s41419-024-07136-0)

Original western blots

USP7 depletion potentiates HIF2α degradation and inhibits clear cell renal cell carcinoma progression

Rongfu Tu, Junpeng Ma, Yule Chen, Ye Kang, Doudou Ren, Zeqiong Cai, Ru Zhang, Yiwen Pan, Yijia Liu, Yanyan Da, Yao Xu, Yahuan Yu, Qi Zhang, Xiaofan Xiong, Donghai Wang, Jingchao Wang, Xinlan Lu, Chengsheng Zhang

Figure 1d

USP7

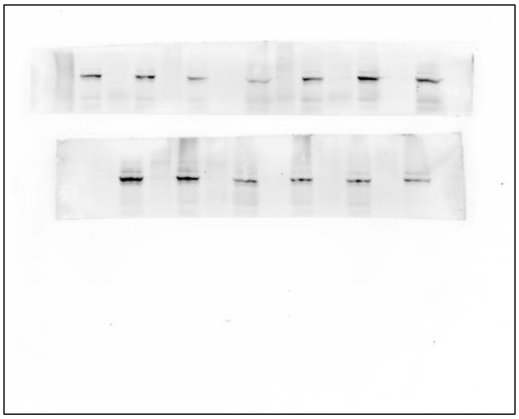

ACTIN

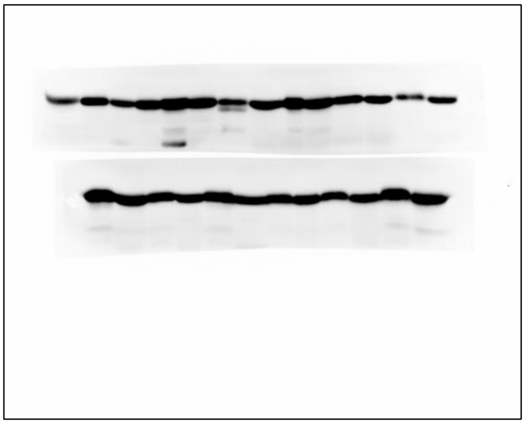

Figure 1g

786-O FUBP1

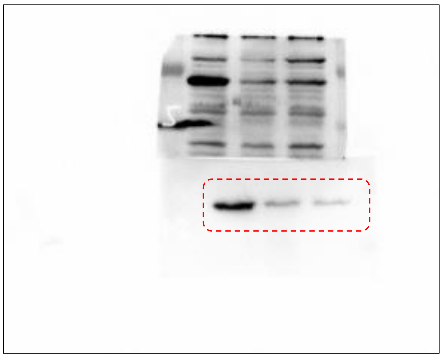

786-O USP7

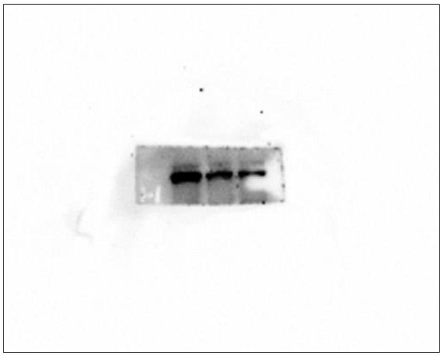

786-O ACTIN

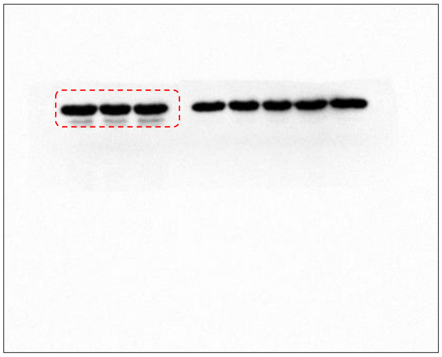

OS-RC-2 FUBP1

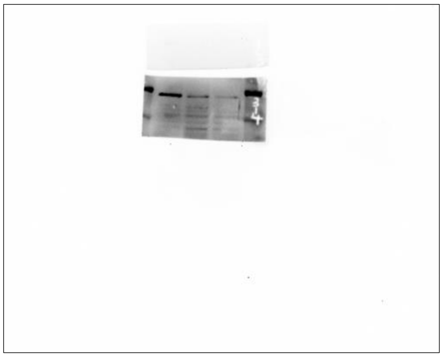

OS-RC-2 USP7

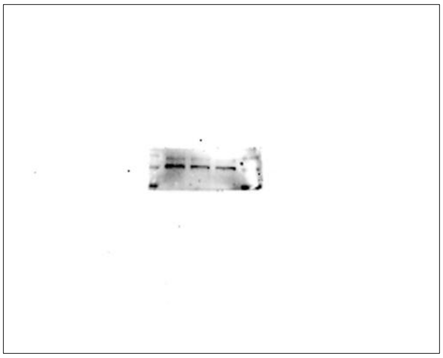

OS-RC-2 ACTIN

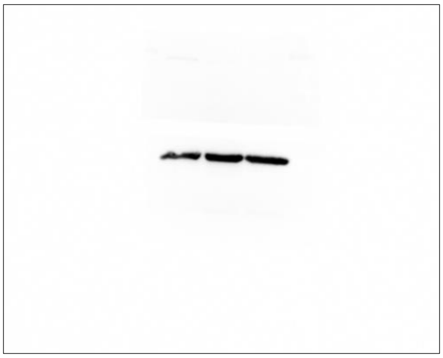

Figure 1h

786-O FUBP3

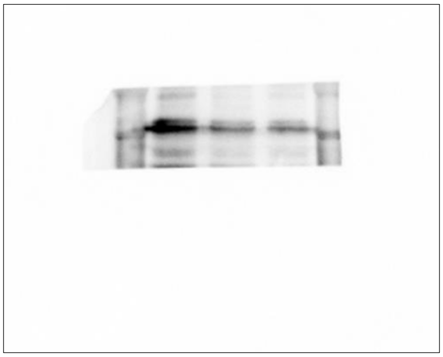

786-O USP7

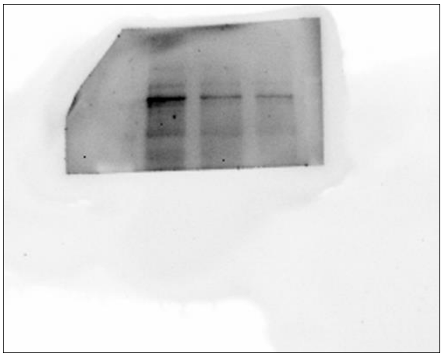

ACTIN

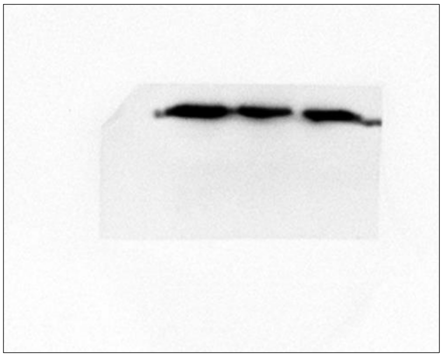

OS-RC-2 FUBP3

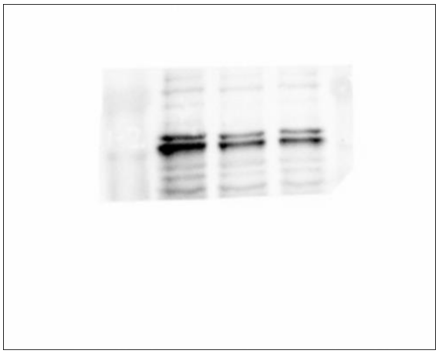

OS-RC-2 USP7

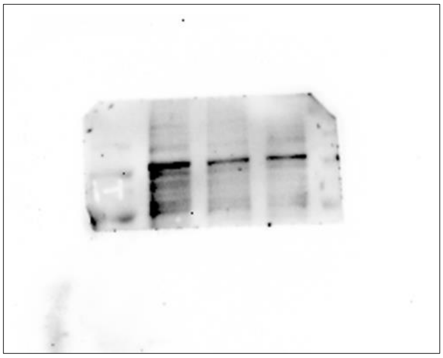

OS-RC-2 ACTIN

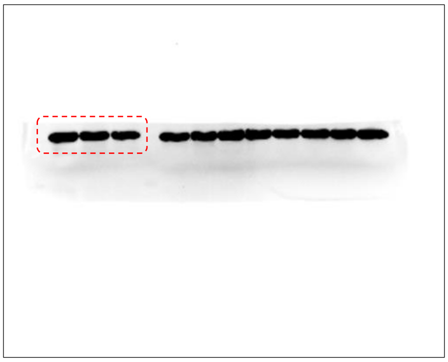

Figure 4a

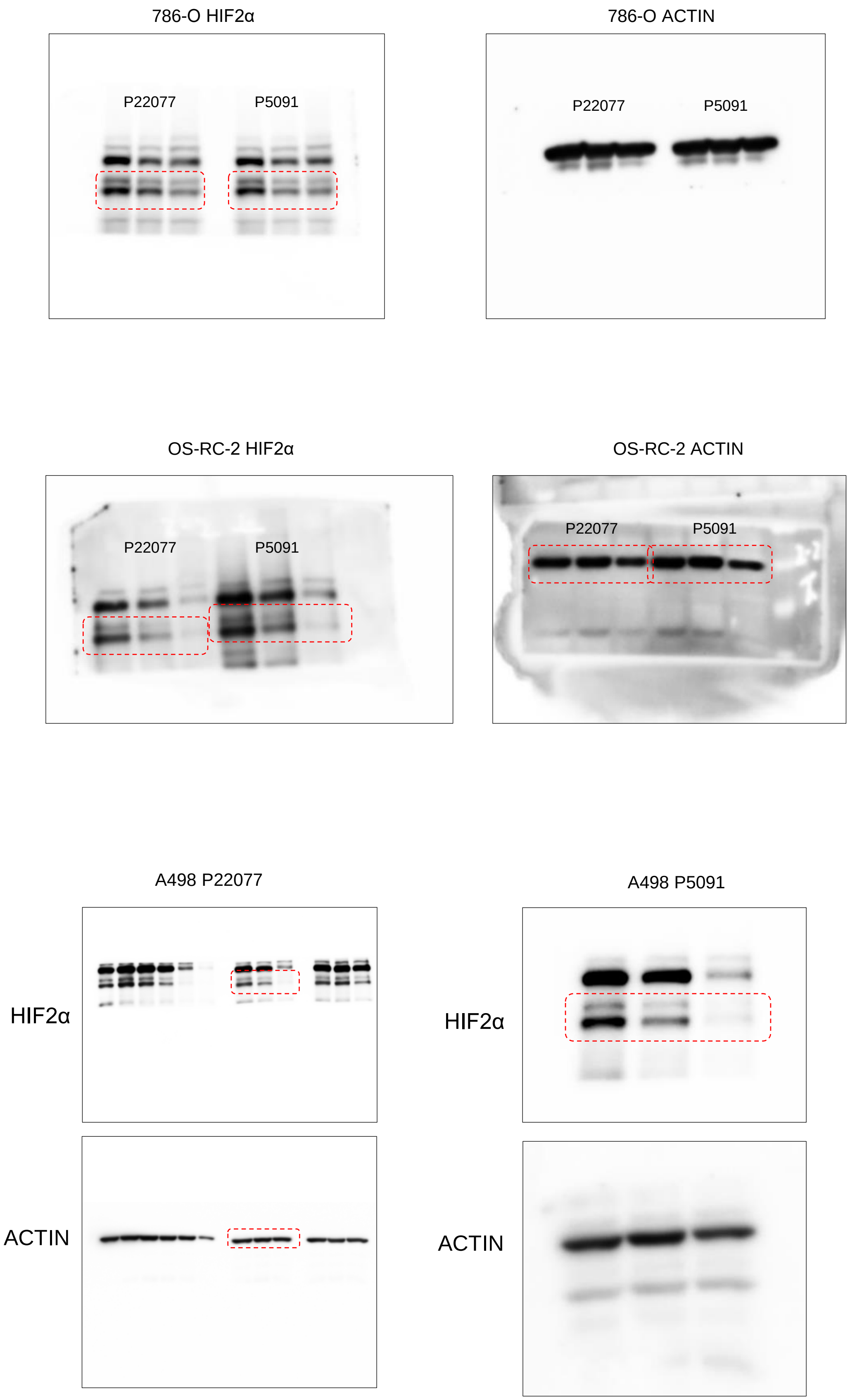

786-O HIF2α
P22077
P5091
786-O ACTIN
P22077
P5091
OS-RC-2 HIF2α
P22077
P5091
OS-RC-2 ACTIN
P22077
P5091
A498 P22077
HIF2α
ACTIN
A498 P5091
HIF2α
ACTIN

Figure 4b

786-O USP7

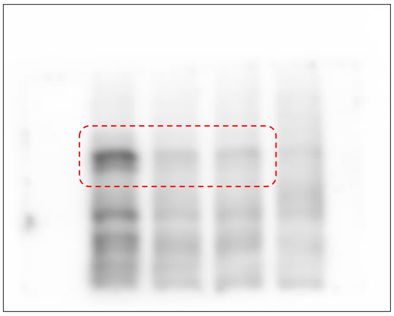

786-O HIF2α

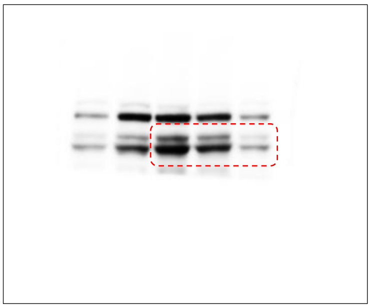

786-O ACTIN

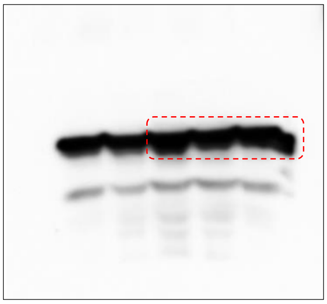

OS-RC-2 USP7

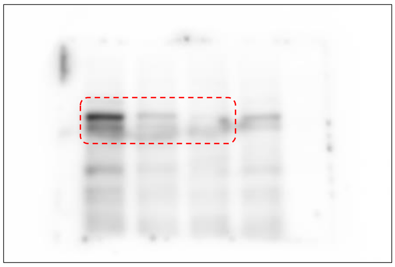

OS-RC-2 HIF2α

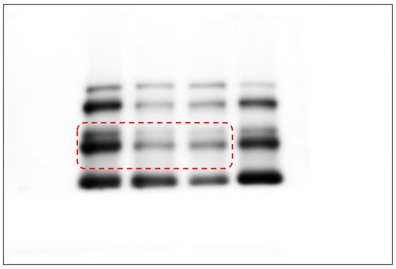

OS-RC-2 ACTIN

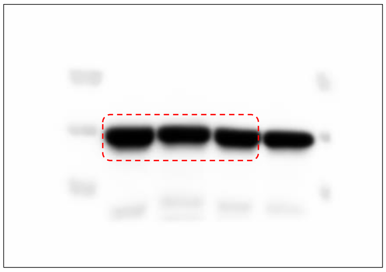

A498 USP7

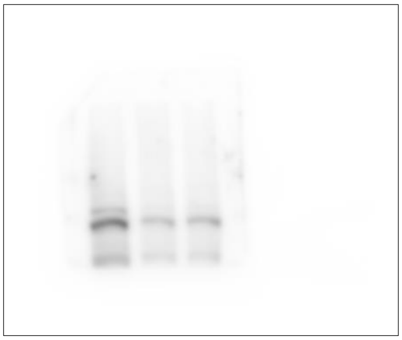

A498 HIF2α

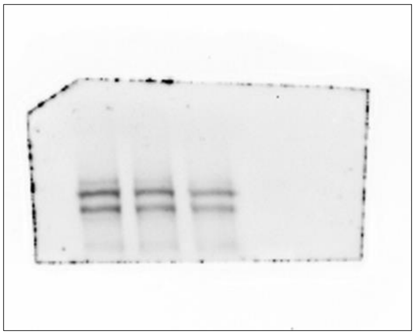

A498 ACTIN

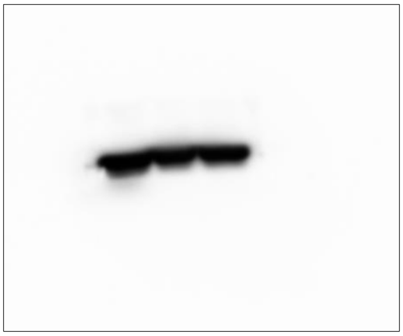

Figure 4c

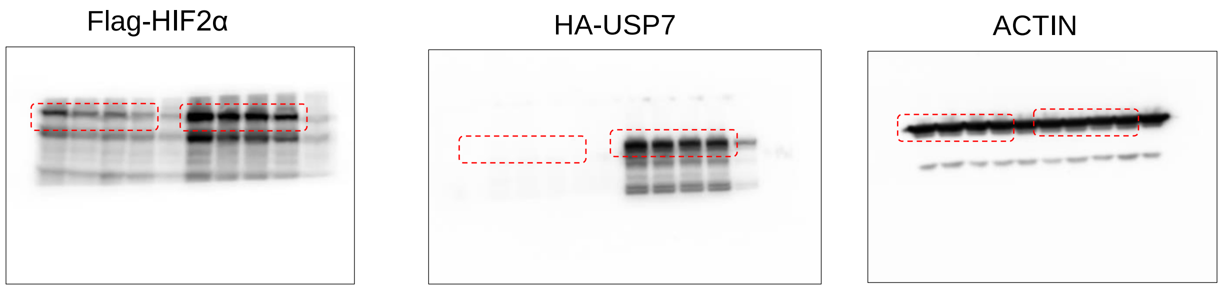

Figure 4d

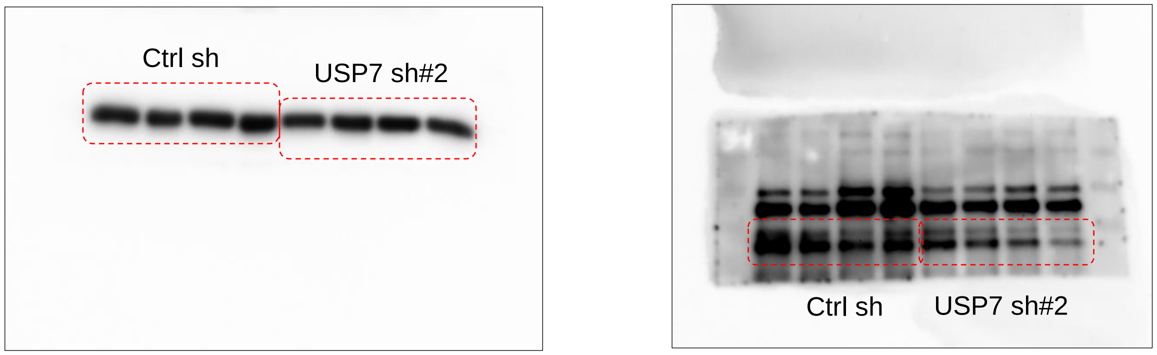

Figure 4e

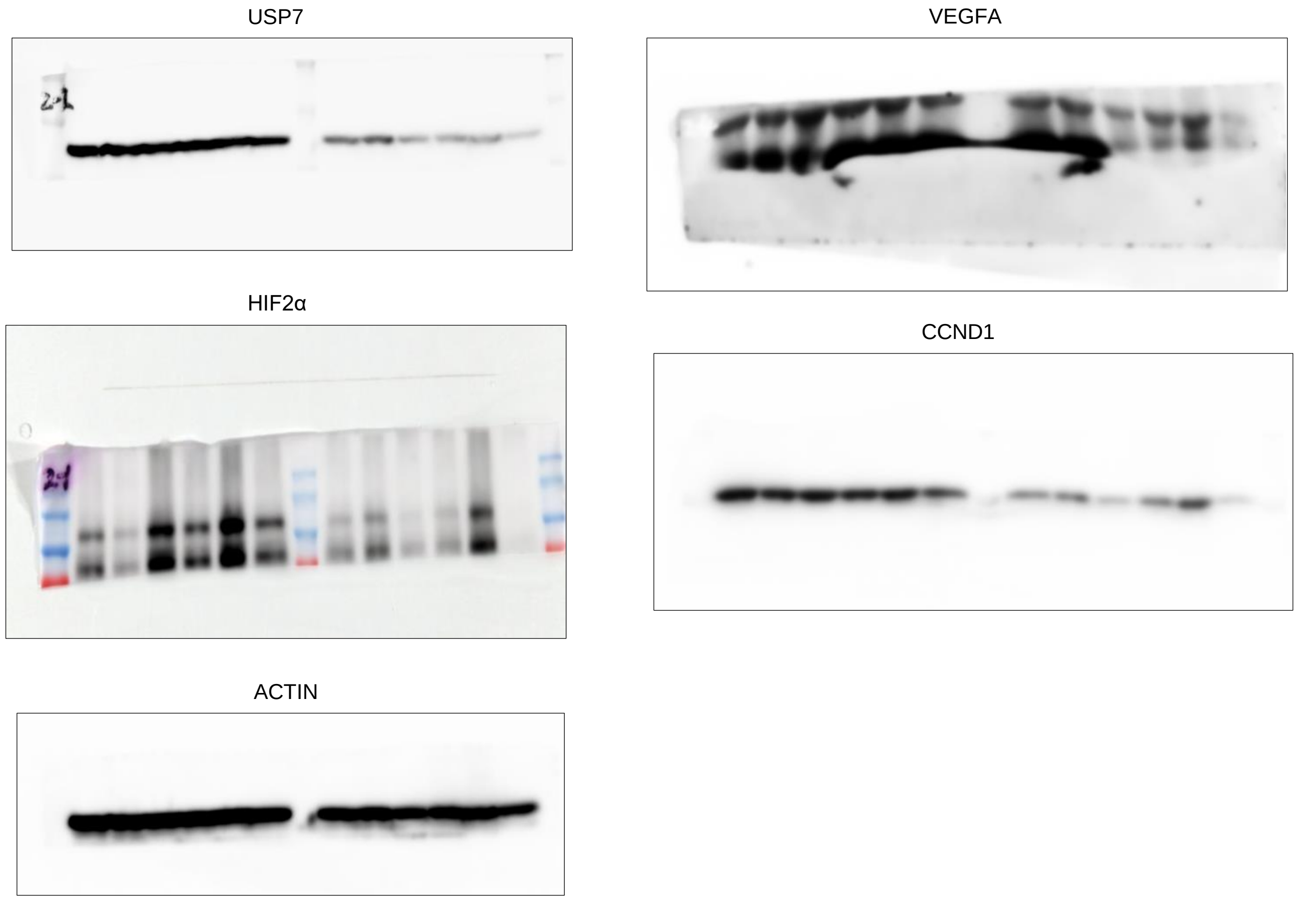

Figure 5a

CO-IP HA-USP7

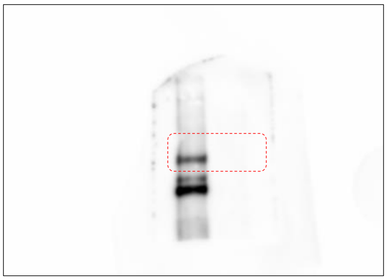

Input HA-USP7

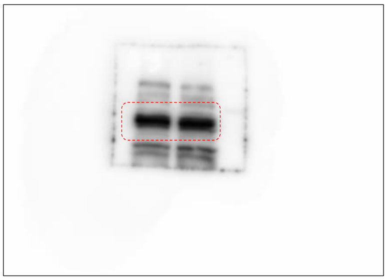

IP Flag-HIF2α

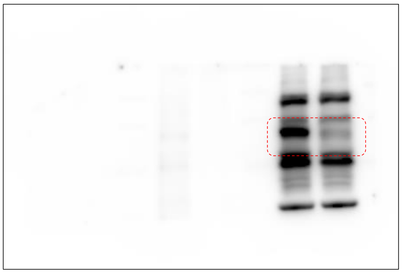

Input Flag-HIF2α

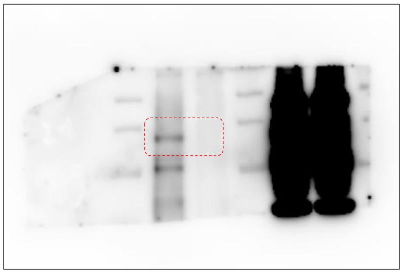

HA-USP7

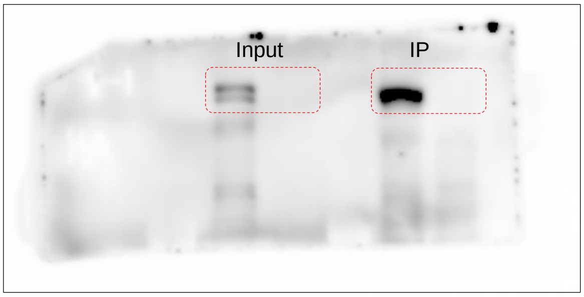

CO-IP Flag-HIF2α

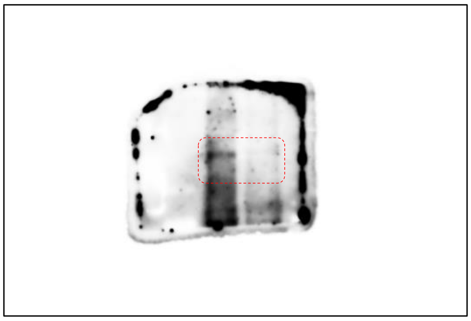

Input Flag-HIF2α

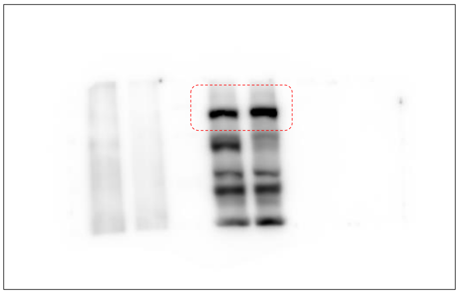

Figure 5b

786-O

IP USP7

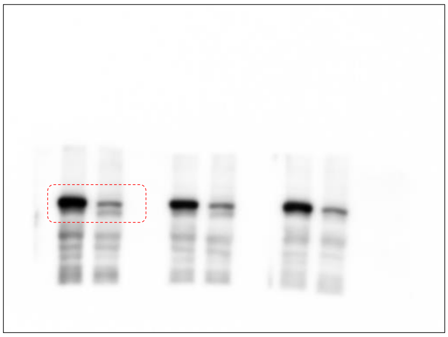

CO-IP HIF2α

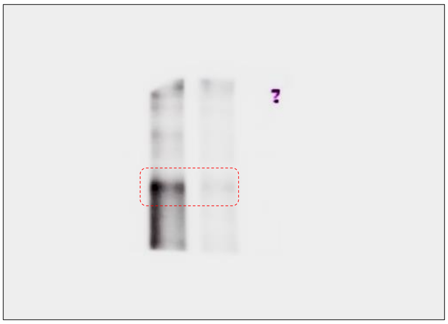

Input USP7

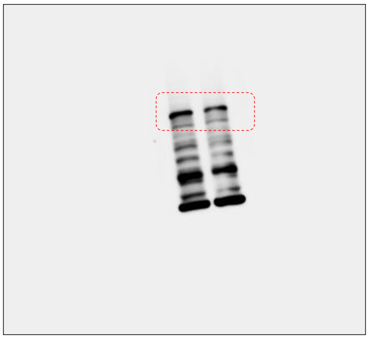

Input HIF2α

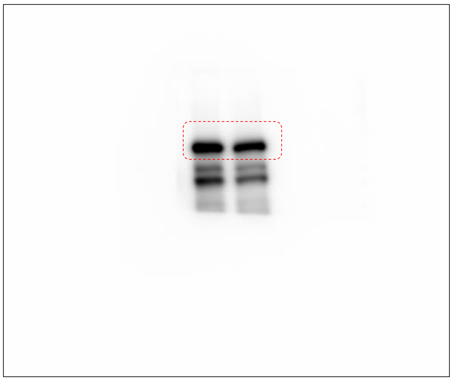

OS-RC-2

IP USP7

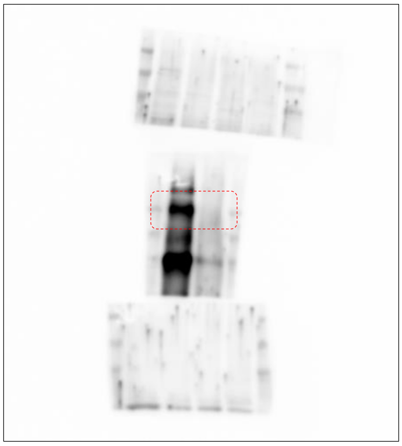

CO-IP HIF2α

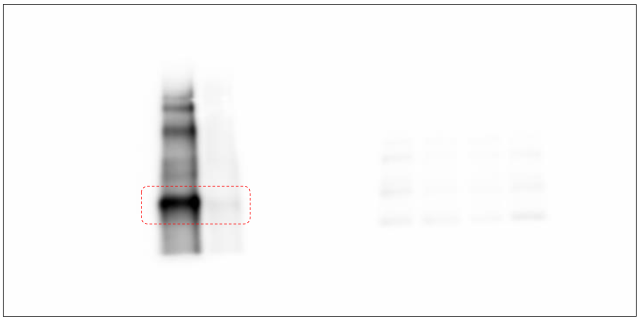

Input USP7

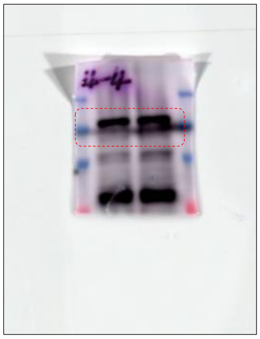

Input HIF2α

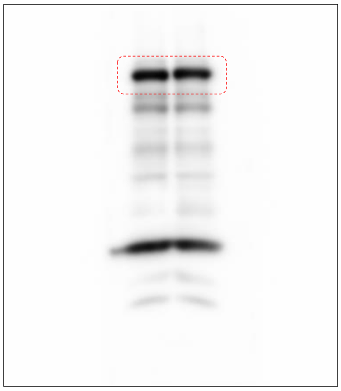

Figure 5c

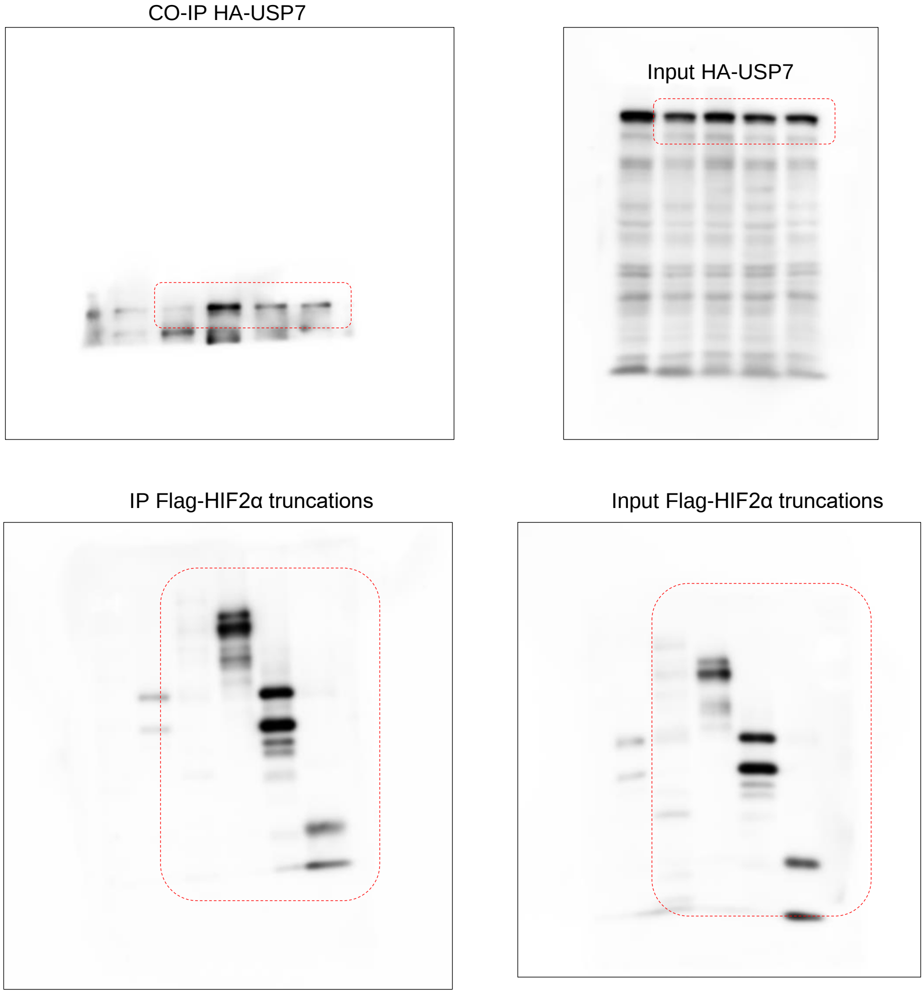

Figure 5d

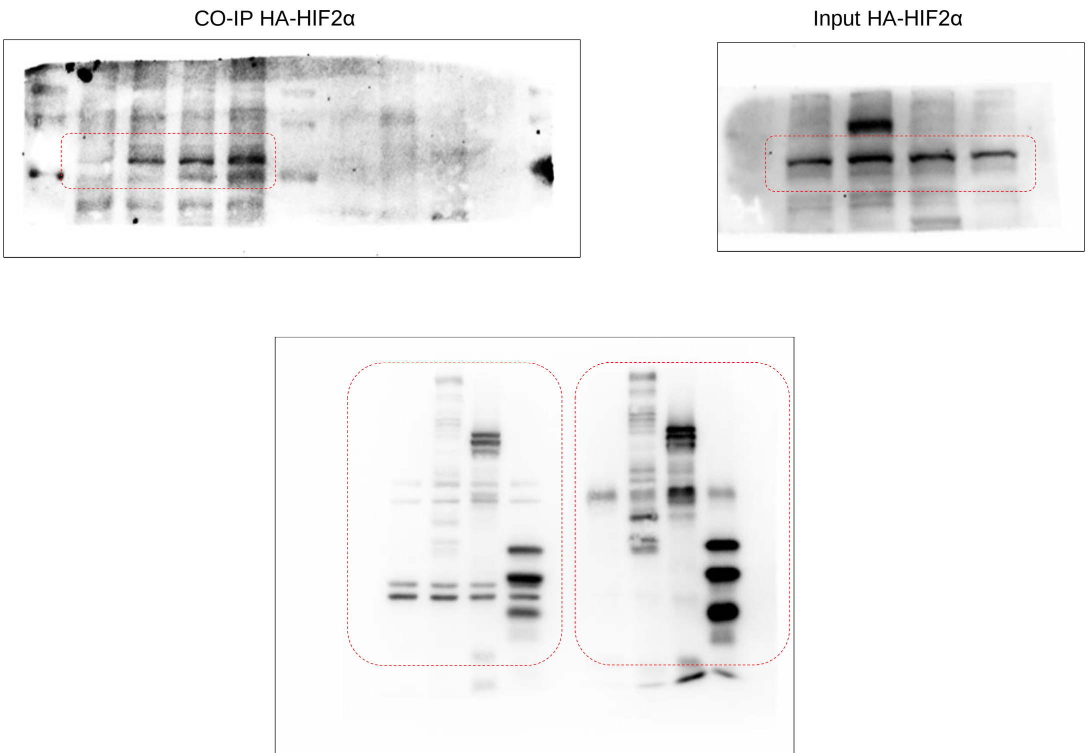

Figure 5e

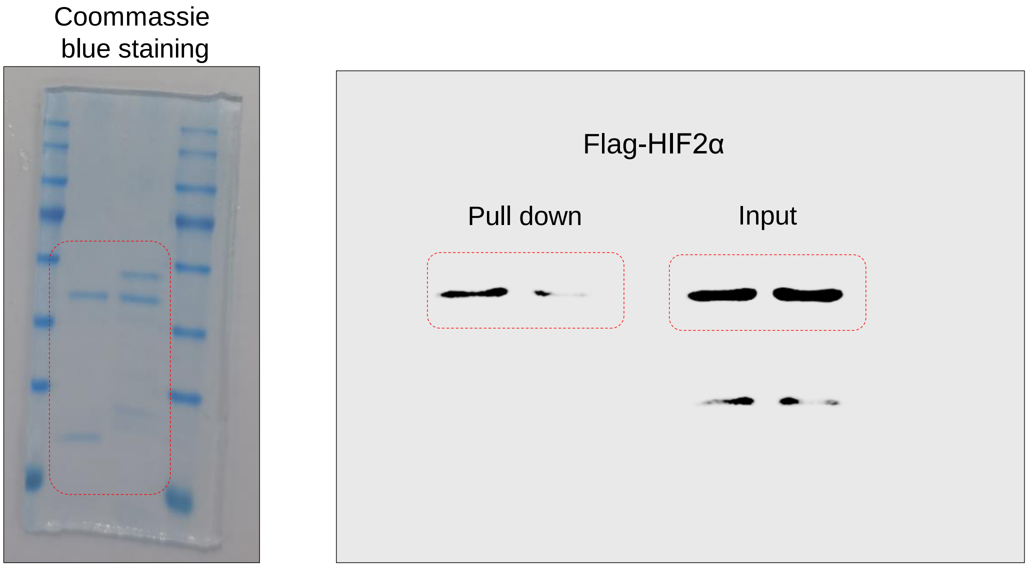

Figure 5f

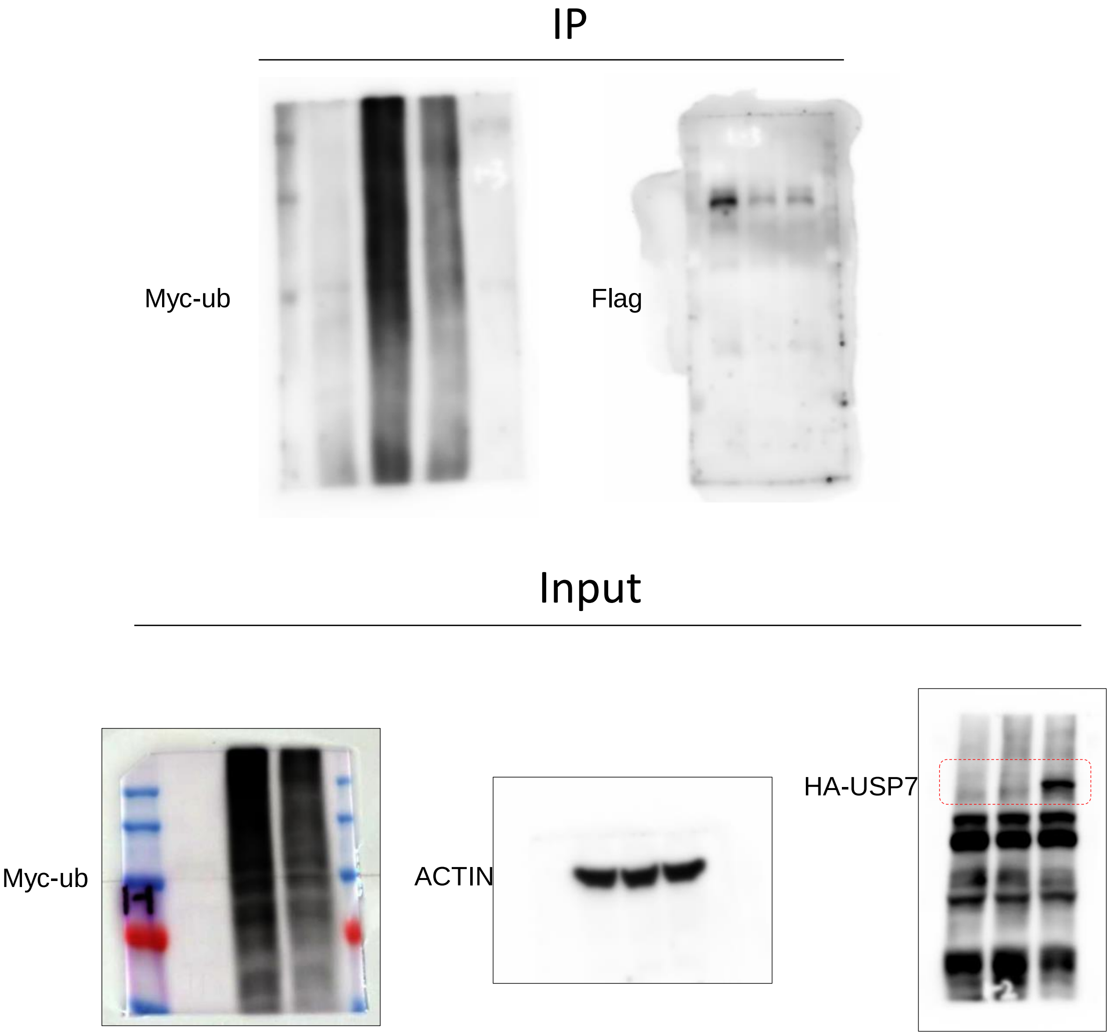

Figure 5g

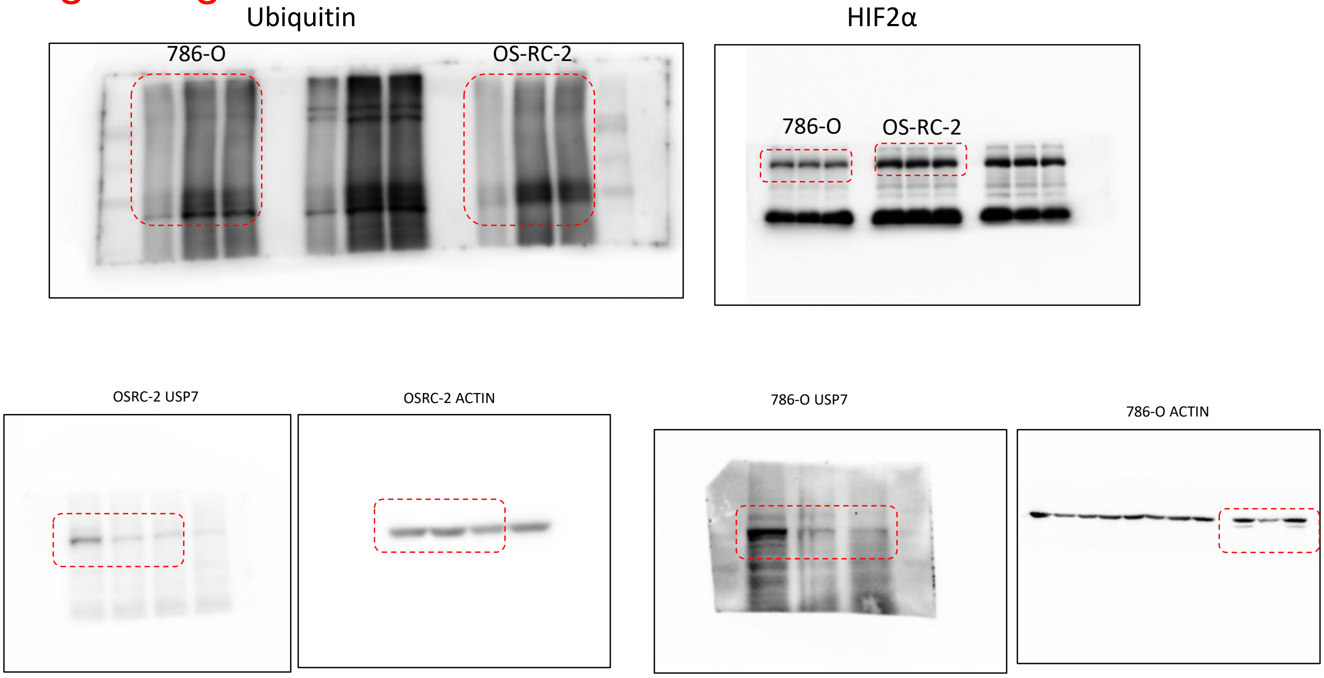

Figure 8a

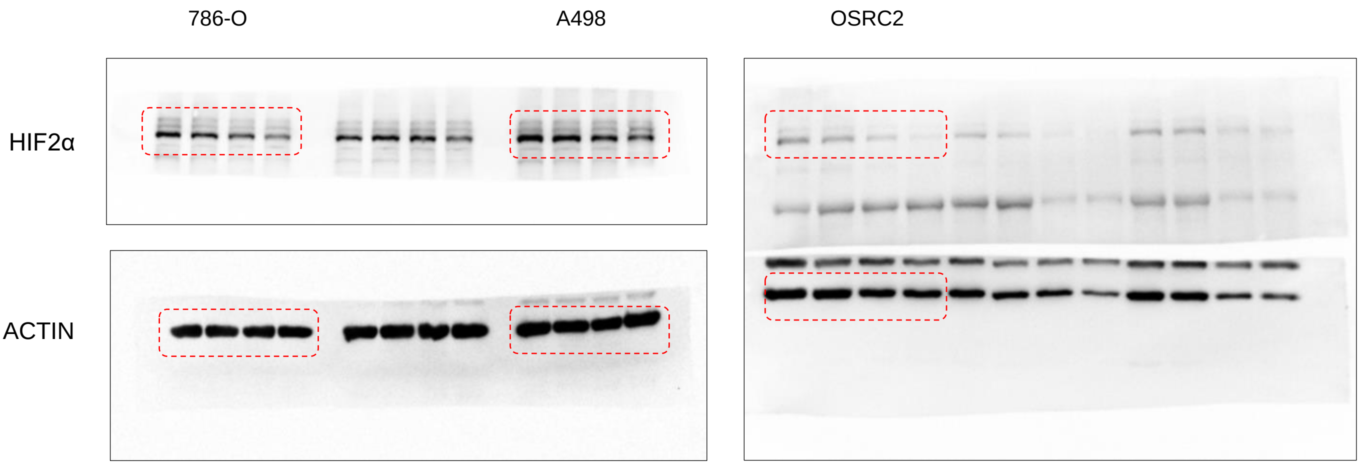

Figure 8b

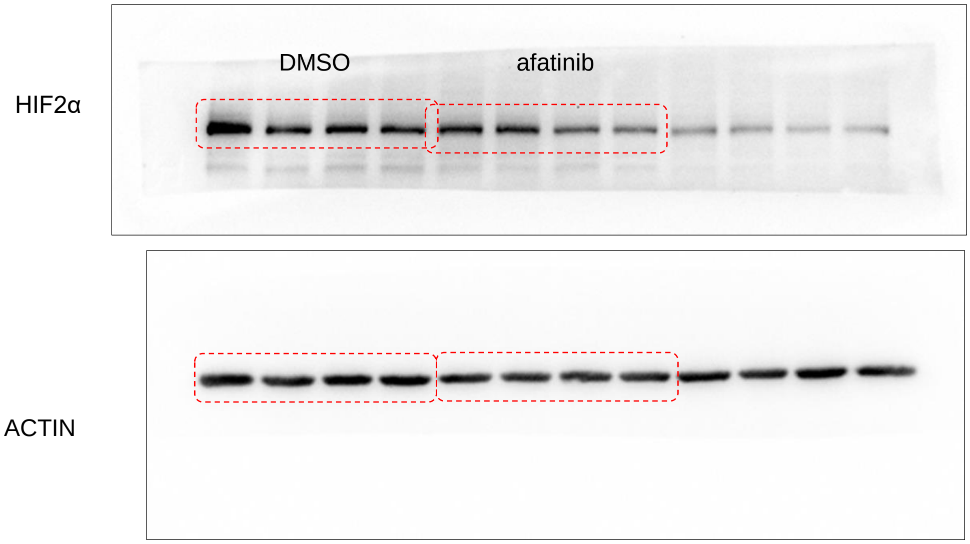

Figure 8c

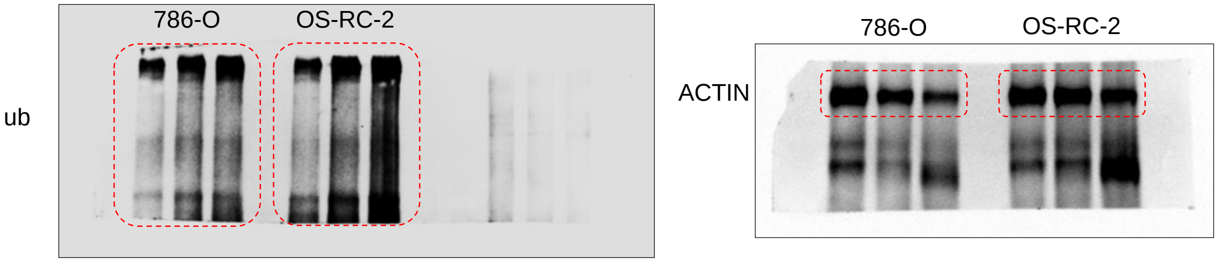

Figure 8e

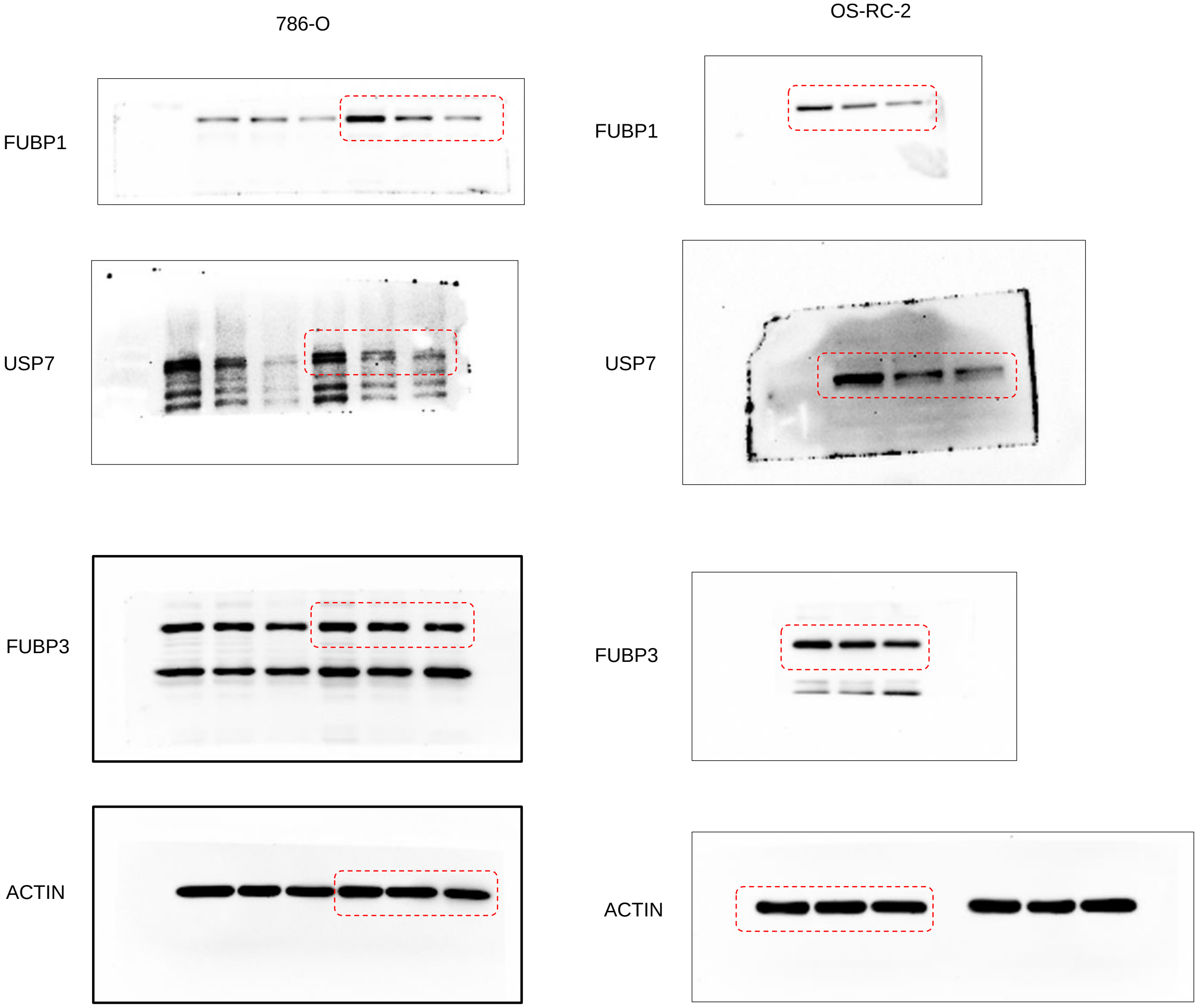

Figure 8f

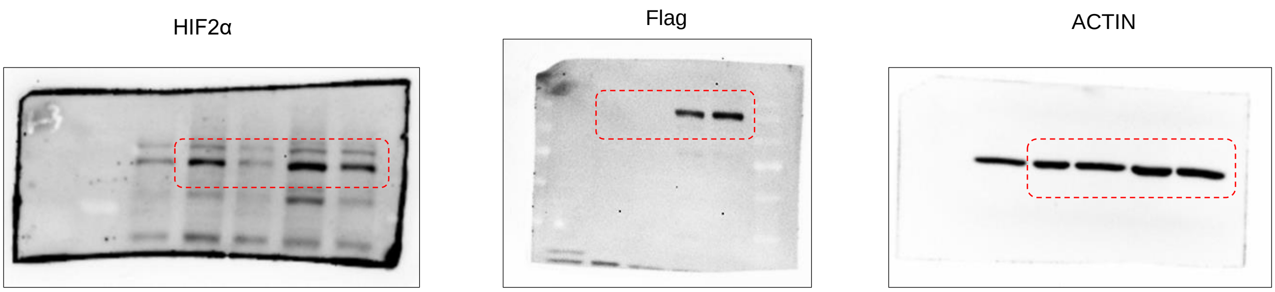

Figure 8g

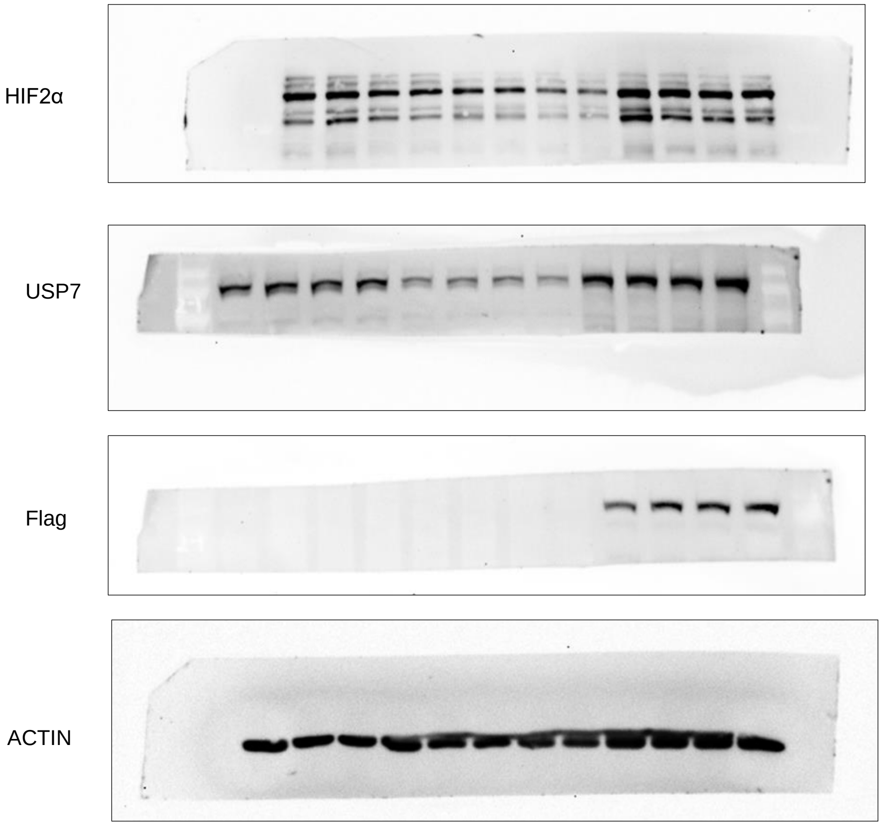

Figure 8h

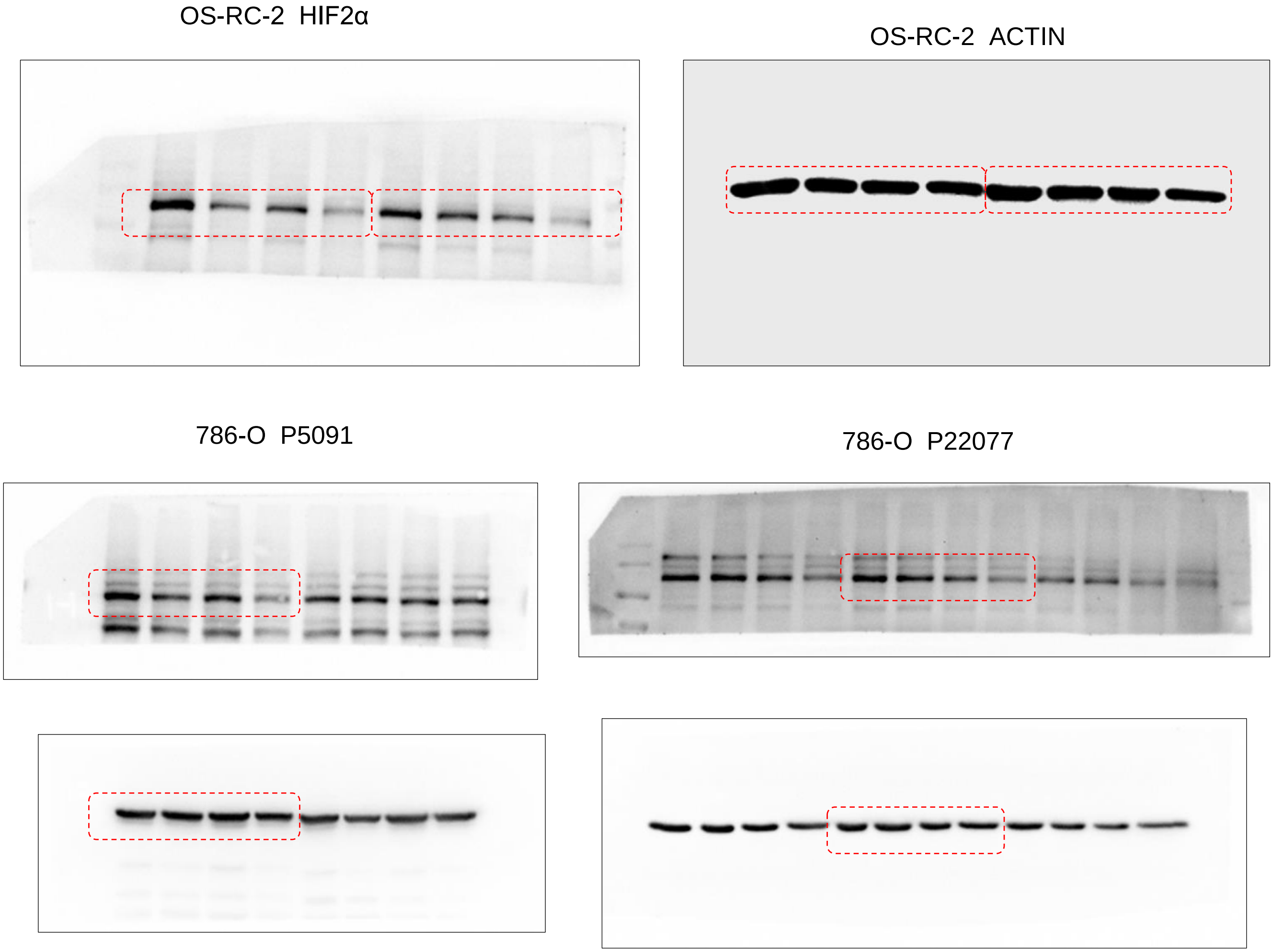

Supplementary Fig 1d

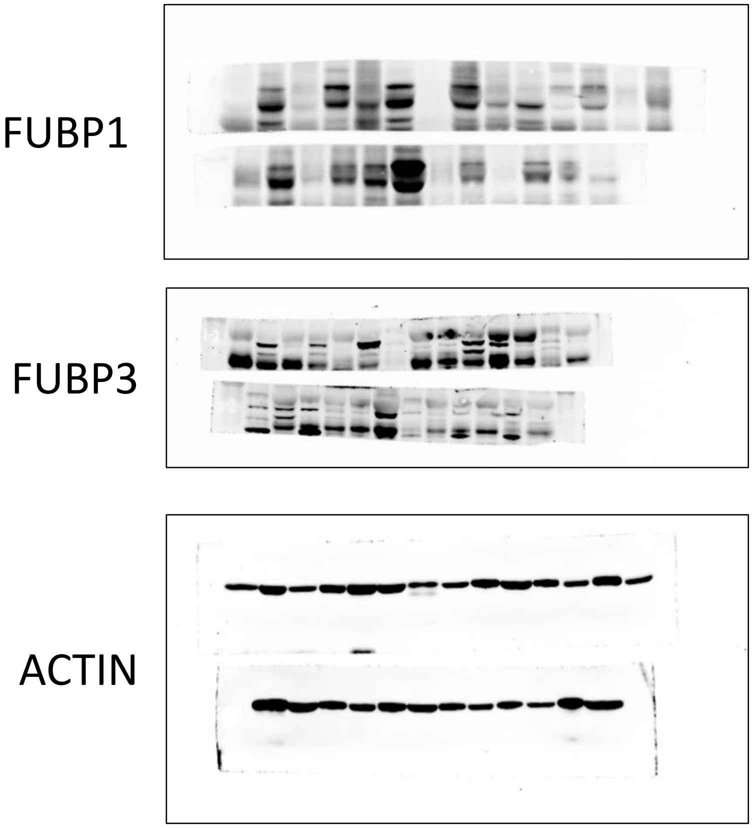

Supplementary Fig 2d

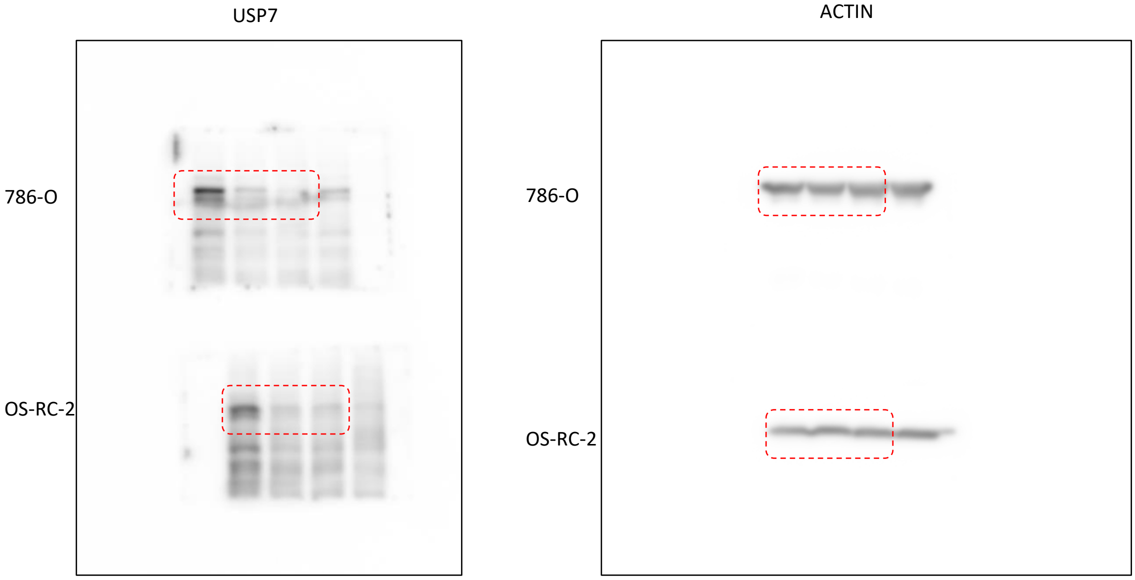

Supplementary Fig 3b

HIF2α

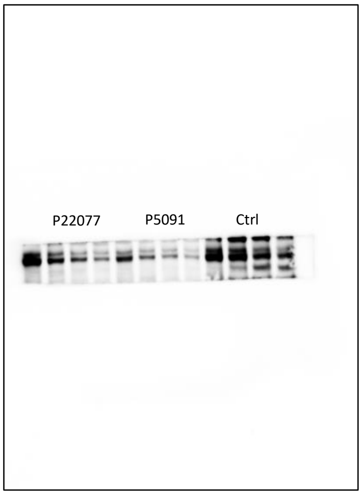

ACTIN

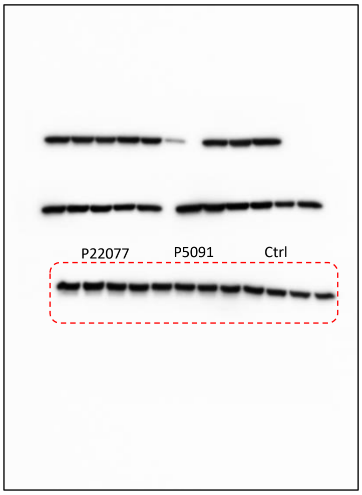

Supplementary Fig 3c

HIF2α

ACTIN

P5091

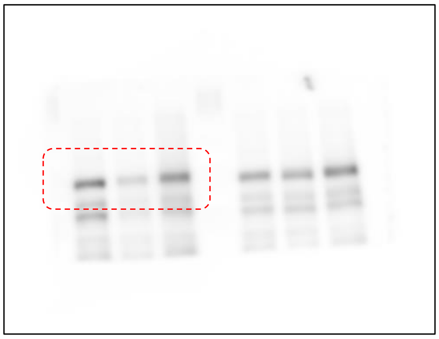

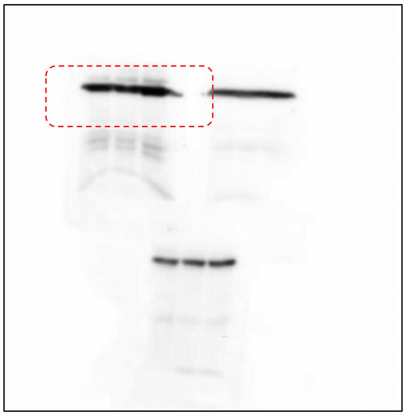

P22077

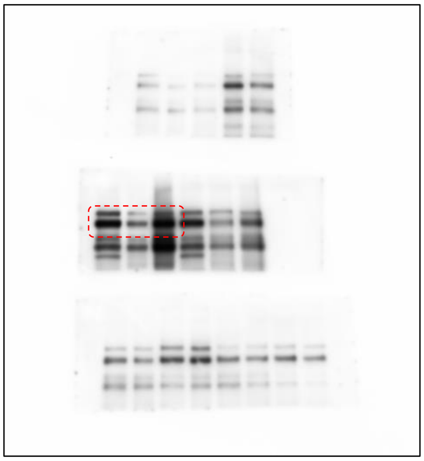

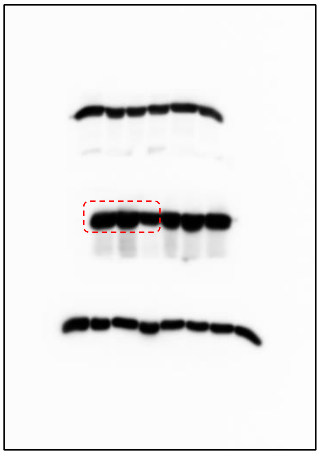

Supplementary Fig 3d

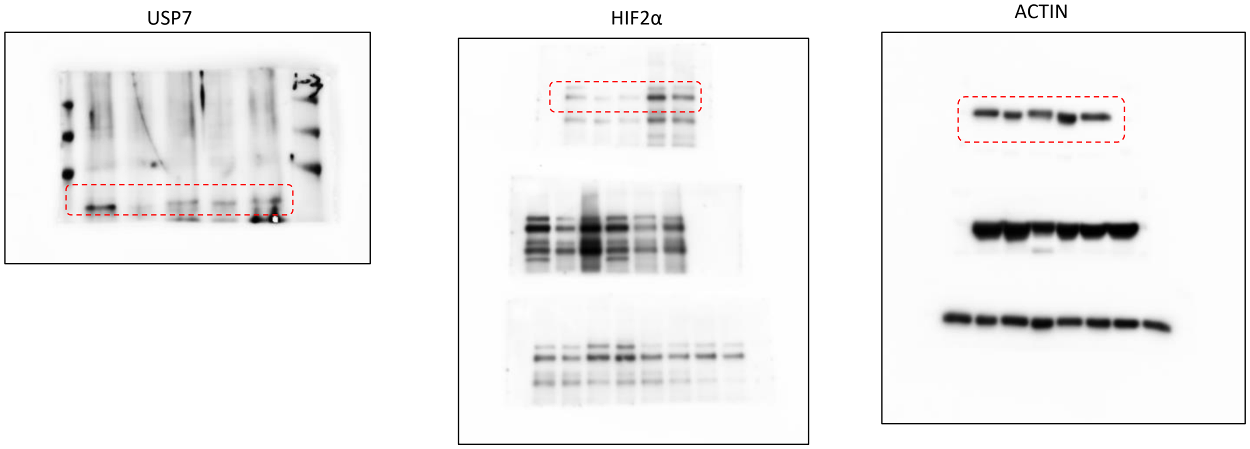

Supplementary Fig 3e

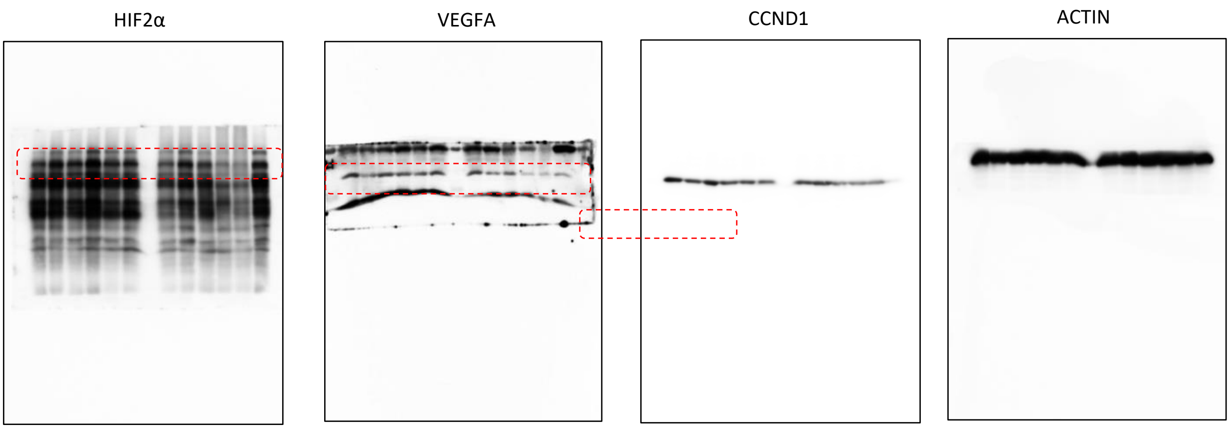

Supplementary Fig 3h

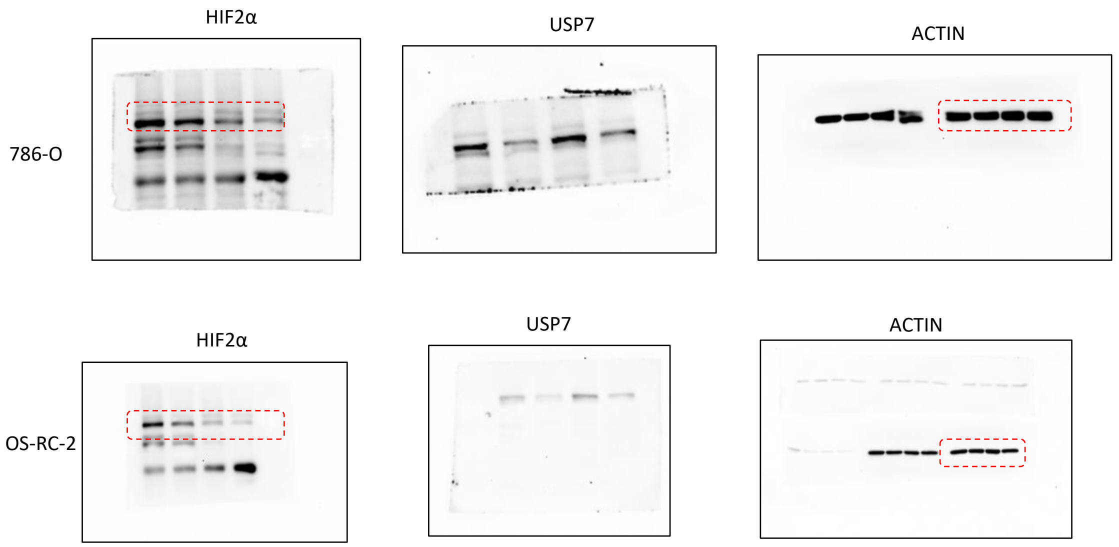

Supplementary Fig 3i

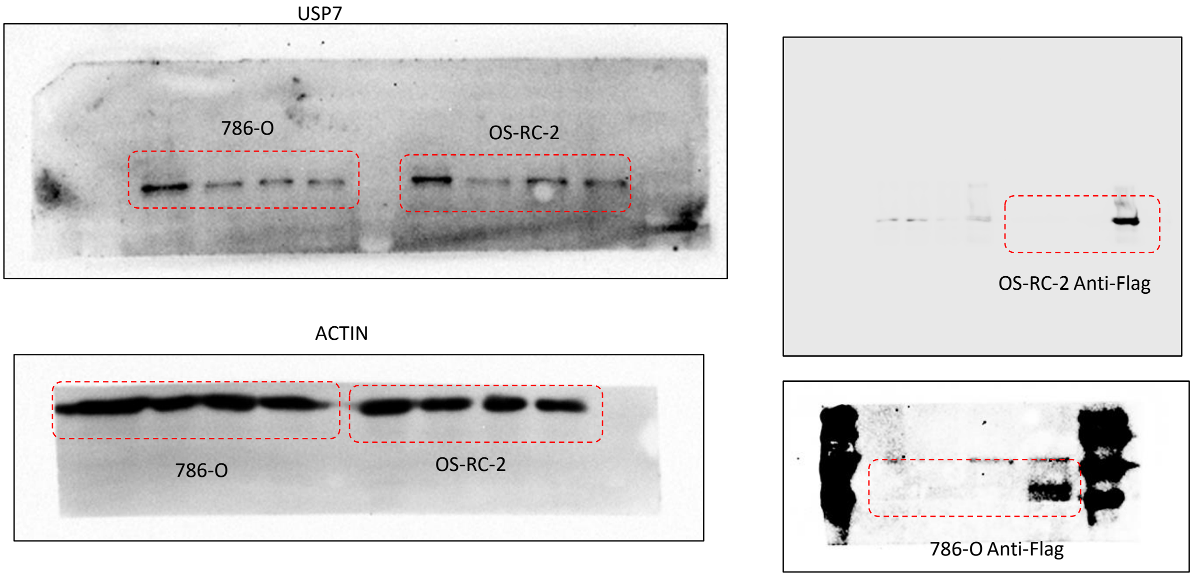

Supplementary Fig 3k

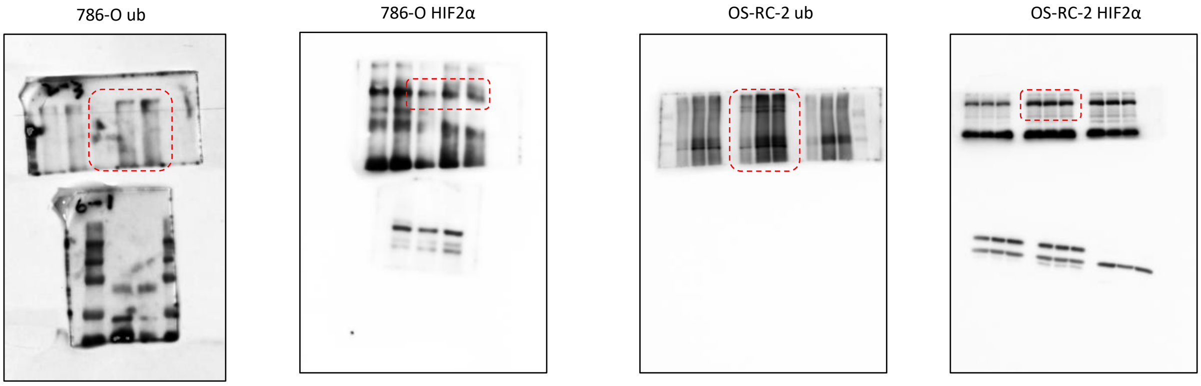

Supplementary Fig 6b

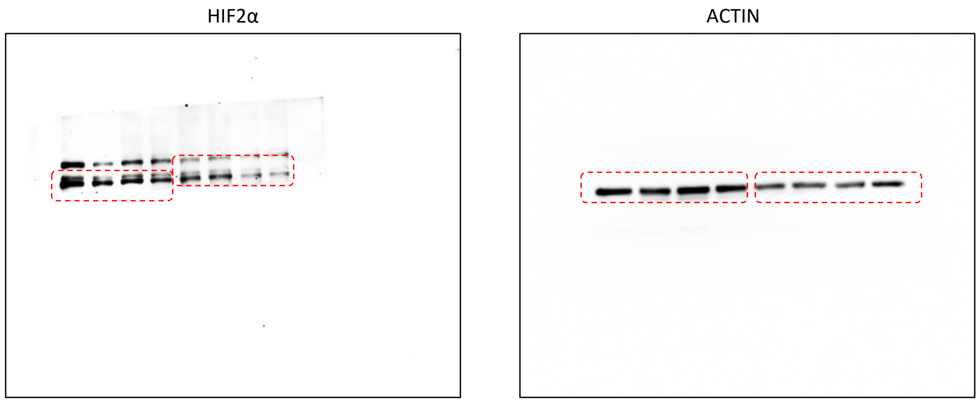

Supplementary Fig 6c

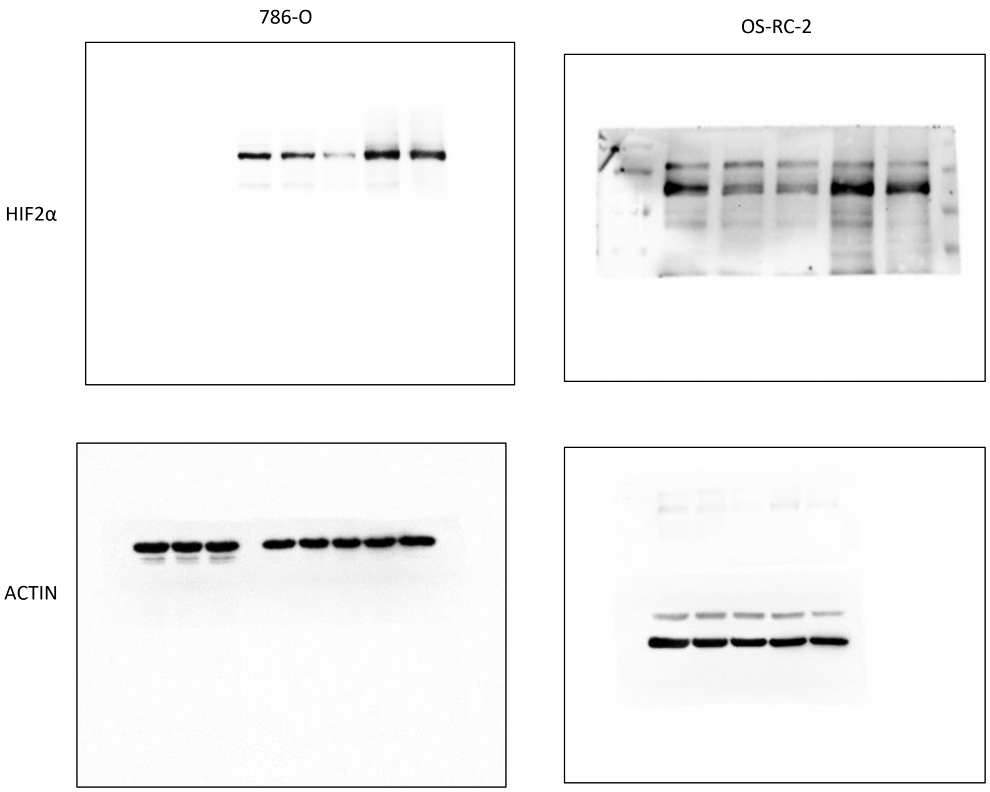

Supplement: Supplementary file 2 — Raw data for Western Blot experiment [file 41419_2024_7136_MOESM2_ESM.pdf]
